# Efficacy and safety outcomes of long-term anti-thrombotic treatment of chronic coronary artery disease: A systematic review and network meta-analysis

**DOI:** 10.3389/fcvm.2022.1016390

**Published:** 2023-01-09

**Authors:** Nayrouz Adawi, Victoria Rotshild, Stav Yanko, Mohammad Mowaswes, Offer Amir, Gal Haitner, Ilan Matok, Bruria Hirsh Raccah

**Affiliations:** ^1^Division of Clinical Pharmacy, Faculty of Medicine, School of Pharmacy, Institute for Drug Research, Hebrew University of Jerusalem, Jerusalem, Israel; ^2^Department of Cardiology, Faculty of Medicine, Hadassah Medical Center, Hebrew University of Jerusalem, Jerusalem, Israel

**Keywords:** network meta-analysis (NMA), chronic coronary syndrome (CCS), dual-anti platelet, anticoagulation, long anti-thrombotic strategies

## Abstract

**Background:**

Clopidogrel, prasugrel, ticagrelor, and low-dose rivaroxaban are all optional strategies in conjunction with aspirin for long-term treatment of chronic coronary artery disease. The aim of this research was to assess the efficacy and safety of long-term anti-thrombotic treatment of chronic coronary heart disease.

**Methods:**

PubMed (MEDLINE), Embase, Clinical Trials Registry ClinicalTrials.gov, and The Cochrane Library were searched through November 2021, to identify randomized controlled trials that compared long term anti-thrombotic therapy for coronary heart disease. Data were extracted to assess eligibility by two independent reviewers. Random-effects meta-analysis was used to pool results.

**Results:**

Eleven randomized controlled trials were included (88,462 patients). In a network meta-analysis, the rivaroxaban compared to the clopidogrel regimen showed lower relative risks (RRs) for death of any cause (0.71; 95% confidence interval [CI], 0.52–0.96), major adverse cardiac events (MACE) (0.73; 95% CI, 0.57–0.93), and cerebrovascular events (0.48; 95% CI, 0.30–0.78). The RR of cerebrovascular events was also lower for the rivaroxaban compared to the ticagrelor 60 mg regimen (0.72; 95% CI, 0.52–0.99). For the prasugrel regimen, the RRs were lower of myocardial infarction incidence versus all extended strategies: clopidogrel plus aspirin (0.76; 95% CI, 0.58–0.99), rivaroxaban (0.60; 95% CI, 0.38–0.93), ticagrelor 60 mg (0.61; 95% CI, 0.42–0.89), and ticagrelor 90 mg (0.63; 95% CI, 0.41–0.97). None of the dual strategies were associated with differences in major bleeding compared to the prasugrel regimen.

**Conclusions and relevance:**

The rivaroxaban regimen appeared to be the preferred long-term anti-thrombotic regimen in preventing all-cause mortality. Our available results tend to support the efficacy of extended anti-thrombotic therapy consisting of prasugrel in lowering MI incidence compared to the other strategies, without increased risk of bleeding. However, additional large-scale direct clinical trials are needed to further determine the adequate long-term anti-thrombotic regimens for treating chronic coronary syndrome.

**Systematic review registration:**

https://www.crd.york.ac.uk/prospero/display_record.php?ID=CRD42020186583, identifier CRD42020186583.

## 1. Introduction

Several studies have described the nature of coronary disease as dynamic, chronic, and progressive, thus indicating its persistence for long and stable periods. On the other hand, the disease may become unstable at any time. The dynamics of the disease lead to a variety of clinical presentations that can be classified into two manifestations: acute coronary syndrome (ACS) and chronic coronary syndrome (CCS) (1). CCS can be modified by controlling risk factors including lifestyle behaviors, by pharmacological therapies for reducing symptoms of angina, and by invasive interventions to attain a stabilized or regressed disease.

Current recommendations suggest considering long-term anti-thrombotic therapy in conjunction with aspirin, for secondary prevention in patients at high or moderate risk for ischemic events (Class IIa and IIb recommendations, respectively, level of evidence A) who are not at a high risk of bleeding. The possible anti-thrombotic therapies are the P2Y12 inhibitors and oral anticoagulants, such as rivaroxaban. The latest guidelines of the European Society of Cardiology on cardiovascular disease prevention strengthen these recommendations (2). Treatment extended 1 year after myocardial infarction (MI) or percutaneous coronary intervention (PCI) has shown benefit mostly by reducing spontaneous MI incidence, which is associated with a 15% increase in mortality rates. However, continued antiplatelet therapy is associated with increased bleeding risk (3). Hence, patients suitable for long-term management should be carefully selected, as indicated in the guidelines. Yet, the efficacy and safety of these anti-thrombotic strategies have not been compared; and to the best of our knowledge, no relevant network meta-analysis (NMA) has been published. In the absence of head-to-head data, the comparative efficacy and safety of P2Y12 inhibitors and low-dose direct oral anticoagulants for reducing long-term ischemic risk remains unknown. This is particularly in regard to high-risk subgroups such as patients with prior MI and prior PCI. Most of the relevant studies were conducted in patients with ACS and CCS along a non-extended phase. While the lines of treatment for selecting appropriate antiplatelet therapy are known for ACS, they are less clear for CCS. Thus, the aim of this study was to compare data on long-term antiplatelet therapies for CCS, in the hope that the results may facilitate knowledgeable clinical decisions, personalized to patients.

## 2. Methods

### 2.1. Search methods for identification of studies

This is a systematic review and meta-analysis of randomized controlled trials (RCTs) that compared long-term anti-thrombotic treatments for chronic coronary syndromes. The literature search and review, and the analysis were performed following the *Preferred Reporting Items for Systematic Reviews and Meta-Analysis* (PRISMA-NMA) 2015 (4). PubMed (MEDLINE), Embase, Clinical Trials Registry ClinicalTrials.gov, and The Cochrane Library were searched until April 2020 and November 2021 in a first and second search, respectively.

The search strategies incorporated index terms (MeSH/Emtree) and text words for the search concepts, while using PI (population and intervention, respectively) categorization. We used wide-ranging search terms and keywords. The detailed search terms and keywords are available in the Supplementary Table 1. No language or date restrictions were applied. The review protocol was registered at the PROSPERO international prospective register of systematic reviews (CRD42020186583). Ethical approval and informed consent were deemed to be exempt as the study consisted of pooling and analyzing already reported data.

### 2.2. Inclusion and exclusion criteria

The criteria for including studies in this NMA were as follows:

1. The inclusion of patients over age 18 years who received anti-thrombotic therapy for more than 12 months and who were diagnosed with coronary heart disease including: ST-elevation myocardial infarction (STEMI), non-STEMI, unstable angina, and stable angina; or who underwent PCI. Articles on *post hoc* and subgroup analysis of included studies were also eligible if they reported predefined outcomes.
2. Descriptions of either dual anti-platelet therapies (clopidogrel, prasugrel, or ticagrelor, plus aspirin) or a combination of an anticoagulant therapy with aspirin (i.e., rivaroxaban plus aspirin); either compared to each other or to a placebo, for prevention of recurrent ischemic heart disease or to reduce the risk of cardiovascular events.
3. Clinical outcomes that included: all-cause mortality, cardiovascular death, MI, or stroke; and major bleeding, with sufficient data in the original studies.

Trials with a treatment duration of less than 12 months or that included patients who received anticoagulant therapy for another indication were excluded. Moreover, we excluded from the analysis non-randomized trials, studies that did not research the question of interest, studies without results or with the wrong intervention, duplicated articles, cohort studies (prospective and retrospective), case-control studies, case series, pharmacokinetic studies in healthy adults, surveys and reviews, expert opinion, editorials, letters to the editor, and comments.

### 2.3. Data extraction and quality assessment

The literature search and study selection were performed by NA and GH independently. Any discrepancies were resolved through consensus or by referring to BR. Stages of literature screening, data extraction, and quality assessment were examined by NA and BR.

The titles and abstracts of all the articles retrieved were screened using Rayyan QCRI (5) application to determine whether they met the inclusion criteria outlined above. Full texts of these potentially eligible studies were retrieved and further assessed for eligibility. A manual search was performed of reference lists of review articles and original studies, to identify additional reports. For the included studies, the following data were extracted: study details (trial registration number, identifier, study design, year of publication, geographical location, and length of follow-up), participant details (number of participants, study population, age, and gender), intervention details (drug type, dosage regimen, and duration of treatment), comparator intervention details, details about primary outcomes (raw data and estimated effect size), and covariate adjustments.

### 2.4. Outcomes

The primary efficacy outcome was the composite of major adverse cardiovascular events (MACE) as calculated by totalizing three incidence rates from each trial: all-cause mortality, MI, and stroke. Secondary efficacy outcomes were individual components of the primary outcome, and also stent thrombosis, which included definite or probable thrombosis according to individual trial definitions and criteria from the Academic Research Consortium (6).

The primary safety outcome was major bleeding. The secondary safety outcome was intracerebral hemorrhage (ICH). For one of the studies, these data were not available, and we included hemorrhagic stroke events under the outcome of ICH (7). Major bleeding was defined according to the definitions used in the individual trials. We presented the definitions of the outcomes that were available from each trial, in the Supplementary Table 2.

We contacted the authors of the DAPT study (8) and requested the outcomes of stroke and all-cause mortality stratified by type of P2Y_12_ inhibitor.

### 2.5. Risk of bias (quality) assessment

The quality of the included studies was assessed using the Cochrane Collaboration tool for assessment of risk of bias for RCTs (9). This included examining random sequence generation, allocation concealment, blinding of participants, personnel and outcome assessors, incomplete outcome data, and selective reporting. Disagreements were resolved by consensus.

### 2.6. The strategy for data synthesis

According to the PRISMA-NMA 2015 (4) we pooled direct and indirect comparisons between the treatment strategies, regarding their relative efficacy and safety. Treatment efficacy and safety outcomes were ranked using the P-score derived from network point estimates. A high P-score value indicates better efficacy and safety of the treatment regimen. The P-score is a frequentist equivalent to the Bayesian network surface under the cumulative ranking curve. The P-score of intervention can be interpreted as the mean extent of certainty that one intervention is better than another intervention. The P-score can be used to rank an intervention within a range of interventions, measured on a scale from 0 (worst) to 1 (best) (10). We incorporated raw data of each outcome as reported in each study. Relative risks (RRs) and 95% confidence intervals (CI) were calculated for indirect comparisons between the strategies regarding their relative efficacy or safety, using the pairwise method.

The analyses and network graphs were performed and generated using frequentist methods in the package “netmeta” within the R environment version 3.4.3. Heterogeneity was interpreted by the τ2 and I^2^ statistic. Inconsistency was assessed by using the Q statistic. We considered evidence of inconsistency if P values were less than 0.05. Subgroup analysis evaluating double blinded RCTs was conducted to assess whether the results of the study were affected by the study design.

We assessed potential publication bias by ‘Comparison-adjusted’ funnel plot in Package “netmeta” for mortality and MI endpoint outcomes. Egger’s test for funnel plot asymmetry is conducted, non-significant p-value indicated symmetrical plot (11).

## 3. Results

### 3.1. Literature search

**FIGURE 1:**
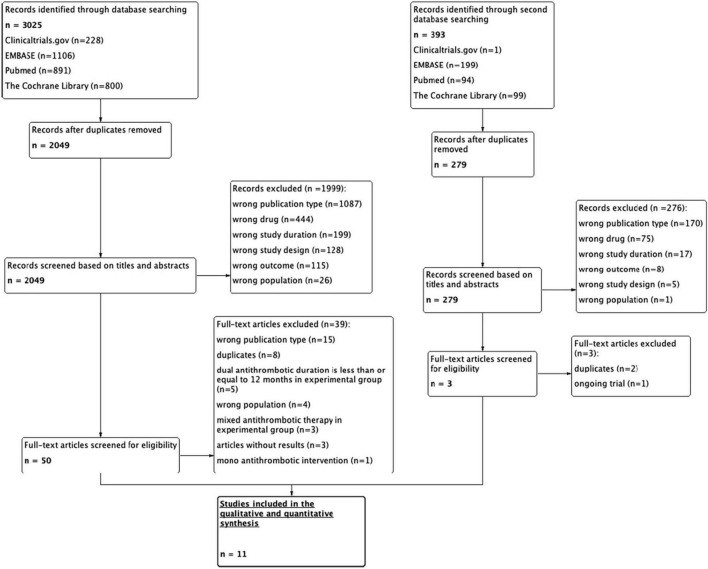
A flow diagram of the selection of studies for the network meta-analysis.

Our broad systematic search yielded 3025 and 393 citations at the first and second searches, respectively. After duplicate articles were removed, 2,049 and 279 articles were left from the respective searches (Figure 1). The process of screening the titles and abstracts according to the data-extraction protocol yielded 50 and 3 articles from the respective searches. These 53 articles underwent full-text review for relevant data of long-term anti-thrombotic treatment for CCS. Ultimately, eleven RCTs (7, 8, 12–20) met the inclusion criteria, of which six were of open-label design (7, 16–20) and five were double-blinded trials (8, 12–15). In total, 88,462 patients were included in this NMA: 16,664 (18.8%) were treated with ticagrelor 60 mg plus aspirin, 14,088 (15.9%) with clopidogrel plus aspirin, 8,313 (9.4%) with rivaroxaban plus aspirin, 7050 (8.0%) with ticagrelor 90 mg plus aspirin, and 5,362 (6.1%) with prasugrel plus aspirin.

In 10 studies, the comparator was aspirin, of which four (8, 13–15) were placebo-controlled trials. Only one study (12) was a head-to-head direct comparison between two P2Y_12_ inhibitors. Most of the studies (7, 8, 16–20) recruited patients who were eligible for PCI and continued with long-term dual therapy throughout the non-acute chronic phase. Three studies (13–15) were designed at the onset to include patients at the chronic phase of coronary disease, indicating stable disease. The mean age of the study participants was 63.2 years. The follow-up period ranged from 18 to 42 months. Forty-seven percent of the patients had diabetes mellitus. Three trials did not report data regarding the multiple vessels involved (8, 12, 20). More than 45% of the patients had multiple coronary artery diseases, and most were at high risk of thrombotic events. Risk factors for atherosclerotic cardiovascular disease, such as hypertension and dyslipidemia, were common. Table 1 shows key characteristics of the included trials. Baseline patients’ characteristics, clinical presentation, and the medical therapy used in each included trial are presented in the Supplementary Tables 3–5.

**TABLE 1:** Characteristics of the included studies and patients.

Study name	RCT Type/ Design	Treatment	Comparator	Population	Total (N)	Mean age (yr)	Male N/(%)	Duration comparison (mo)	Follow up (mo)	Time of randomization	Time from PCI to randomization
**Dapt study**											
Mauri et al. (8)	Double-Blind Superiority	Thienopyridine drug plus ASA (75–162 mg daily)	Placebo plus ASA (75 -162 mg daily)	Patients older than 18 years of age who were candidates for DAPT after treatment with FDA-approved drug-eluting stents	9961	61.7	7435/ (74.64%)	12 vs. 30 = 18	33	12 mo. after PCI	12 mo.
		Clopidogrel 75 mg daily									
NCT00977938		Prasugrel✧10 mg daily									
**Themis**											
Steg et al. (13)	Double-Blind Superiority	Ticagrelor* (60 mg twice daily) plus ASA (75–150 mg daily).	Placebo plus ASA (75–150 mg daily)	Patients who were 50 years of age or older and who had stable CAD (a history of previous PCI or CABG or documentation of angiographic stenosis of at least 50% in at least one coronary artery) and type 2 diabetes mellitus	19220	66	13189/ (68.62%)	Median of 39.9		At enrollment	Median of 3.3 yr.
NCT01991795											
**Compass**											
Connolly et al. (14)	Double-Blind Superiority	Rivaroxaban (2.5 mg twice daily) plus ASA (100 mg once daily)	Placebo twice daily and ASA (100 mg daily).	Patients who were at least 65 years old with a diagnosis of CAD, patients had to have either MI within 20 years, multi-vessel CAD, history of stable or unstable angina, previous multi-vessel PCI, or previous multi-vessel CABG	16574	69	13192 (79.59%)	Mean of 1.95 yr. : 23.4		After a 30-day run-in period since enrollment	Mean of 5.4 yr.
NCT01776424											
**Optidual**											
Helft et al. (7)	Open Label Superiority	Clopidogrel (75 mg daily) plus ASA (75–160 mg daily)	ASA (75–160 mg daily)	Patients had symptoms of stable angina, silent ischemia, ACS (unstable angina, NSTEMI, STEMI) with ≥ 1 lesion with stenosis ≥ 50% located in a native vessel ≥ 2.25 mm in diameter and who were implanted with ≥ 1 DES of any type	1385	64.1	1115/ (80.50%)	12 ± 3 vs. 48 ± 3 = 36	Median of 33.4 (IQR, 18.9–36.5)	12 ± 3 mo. after PCI	12 ± 3 mo.
NCT00822536											
**Pegasus-timi**											
Bonaca et al. (15)	Double-Blind Superiority	Ticagrelor (90/60 mg twice daily) plus ASA (75–150 mg daily)	Placebo plus ASA (75–150 mg daily)	Patients had spontaneous MI 1 to 3 years before enrollment, were at least 50 years of age, and had one of the following additional high-risk features: age of 65 years or older, diabetes mellitus requiring medication, a second prior spontaneous MI, multivessel CAD, or chronic renal dysfunction: defined as an estimated creatinine clearance of less than 60 ml/min.	21162	65.3	16102/ (76.10%)	Median of 33 (IQR, 28 to 37)		At enrollment	Median of 1.6 yr.
		Ticagrelor (90 mg twice daily) plus ASA (75–150 mg daily)									
		Ticagrelor (60 mg twice daily) plus ASA (75–150 mg daily)									
NCT01225562											
Lee et al. (16)	Open Label Superiority	Clopidogrel (75 mg daily) plus ASA (100–200 mg daily)	ASA (100-200 mg daily)	Patients had undergone implantation with DES at least 12 months before enrolment, no MACE (MI, stroke, or repeat revascularization) or major bleeding since implantation, DAPT on board	5045	62.4	3498/ (69.33%)	12 vs. 36 = 24	Median of 42.0 (IQR, 24.7–50.7)	12-18 mo. after PCI	12 mo.
NCT01186146											
**Prodigy**											
Valgimigli et al. (17)	Open Label Superiority	Clopidogrel (75 mg daily) plus ASA (80–160 mg daily)	ASA (80–160 mg daily)	Patients undergoing elective, urgent, or emergent coronary angioplasty with intended stent implantation	1970	67.8	1511/ (76.70%)	6 vs. 24 = 18	24	30 ± 5 days after PCI	30 ± 5 days
NCT00611286											
Dadjou et al. (20)	Open Label	Clopidogrel (75 mg daily) plus ASA (75 mg daily)	ASA (75 mg daily)	Patients who were referred for elective, urgent, or emergency coronary angioplasty with intended stent implantation	1010	60	647 (64.05%)	Less vs. more than 12	More than 36	Randomization at index PCI	
NCT02327741											
**Real-late/Zest late**											
Park et al. (18)	Open Label Superiority	Clopidogrel (75 mg daily) plus ASA (100–200 mg daily)	ASA (100 mg daily)	Patients who had received drug-eluting stents and had been free of major adverse cardiac or cerebrovascular events and major bleeding for a period of at least 12 months to receive clopidogrel plus aspirin or aspirin alone.	2701	61.9	1883/ (69.71%)	12 vs. 36 = 24	Median of 33.2 (IQR, 28.1–37.6)	12 mo. after PCI with the placement of DES	12 mo.
NCT00484926, NCT00590174											
**Smart-date ^&^**											
Hahn et al. (19)	Open Label Non-inferiority	P2Y12 inhibitor** plus ASA (100 mg daily)	ASA (100–200 mg daily)	Patients had unstable angina, NSTEMI, or STEMI, with at least one lesion in a native coronary vessel with reference diameter of 2.25–4.25 mm and stenosis >50% amenable for PCI with stents.	2712	62.1	2044/ (75.36%)	6 vs. 12.6 to 18	Median of 17.7 (IQR, 12.6–18.0)	Randomization at index PCI	
		Clopidogrel (75 mg daily) plus ASA (100 mg daily)			2191^&^		1651/ (75.36%)				
NCT01701453											
**Trilogy^**											
Roe et al. (12)	Double-Blind Superiority	Prasugrel (10 mg daily) plus ASA	Clopidogrel (75 mg daily) plus ASA	Patients (age < 75) with unstable angina or NSETMI who do not undergo revascularization	7243	62	4644/ (64.12%)	6 to 30 months	Median of 17.1 (IQR, 10.4-24.4)	Within 10 days of the index event	Excluded
NCT00699998											

✧ Dose of 5 mg daily recommended in patients who weighed less than 60 kg.

* Patients converted to dosage regimen of 60 mg twice daily after median exposure of 7.7 months to the 90 mg dose.

**Clopidogrel 75 mg daily or prasugrel 10mg daily or Ticagrelor 90 mg twice daily.

& Due to missing data referred to patients randomly assigned to another P2Y_12_ inhibitor separately, only available data on clopidogrel was included in the analysis.

^The comparator in this trial is considered also as intervention.

In the SMART-DATE study (19) data were only available for patients treated with clopidogrel, and not for those randomly assigned to receive another P2Y_12_ inhibitor (19). Randomization was stratified by the type of P2Y_12_ inhibitor; thus, consistent relative percentages were considered.

We compared five treatment strategies: clopidogrel plus aspirin, prasugrel plus aspirin, ticagrelor 60 mg or 90 mg plus aspirin, and rivaroxaban plus aspirin. Supplementary Figure 1 shows the network of treatment regimens used in the analysis of the primary efficacy outcome and the MI outcome.

### 3.2. Efficacy outcomes

#### 3.2.1. All-cause mortality

**FIGURE 2:**
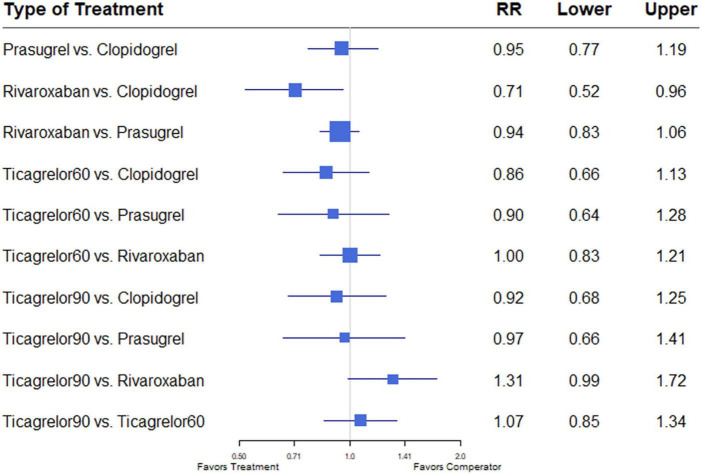
A network meta-analysis of the association between anti-thrombotic therapy and mortality in patients with chronic coronary syndrome. Net-work Meta-analysis of the Association Between anti-thrombotic therapy and mortality. The size of squares is proportional to the weight of each study. Horizontal lines indicate the 95% CI of each treatment; diamond, the pooled estimate with 95% CI; and RR, relative risk.

Of 11 studies, nine (7, 12–18, 20) reported the outcome of all-cause mortality. Overall mortality events in these studies were 3,422 (4.48%) of 76,310 patients. Heterogeneity between the studies when considering all-cause mortality was *I*^2^ = 16%. Compared with the clopidogrel plus aspirin regimen, rivaroxaban plus aspirin significantly reduced all-cause mortality (RR, 0.71; 95% CI, 0.52–0.96) (Figure 2).

#### 3.2.2. MACE

**FIGURE 3:**
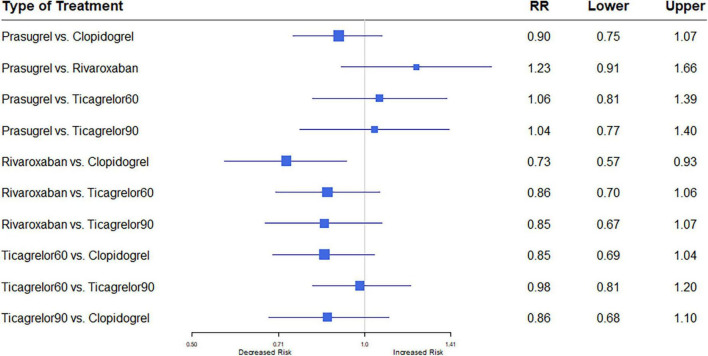
A network meta-analysis of the association between anti-thrombotic therapy and major adverse cardiac events (MACE) in patients with chronic coronary syndrome. Net-work Meta-analysis of the Association Between anti-thrombotic therapy and mortality. The size of squares is proportional to the weight of each study. Horizontal lines indicate the 95% CI of each treatment; diamond, the pooled estimate with 95% CI; and RR, relative risk.

Data extracted from nine studies including 76,310 patients (7, 12–18, 20) reported 7,027 events. Heterogeneity between the studies when considering MACE was *I*^2^ = 34.6%. Compared with the clopidogrel plus aspirin regimen, the rivaroxaban plus aspirin regimen significantly reduced MACE (RR, 0.73; 95% CI, 0.57–0.93) (Figure 3).

#### 3.2.3. Myocardial infarction

**FIGURE 4:**
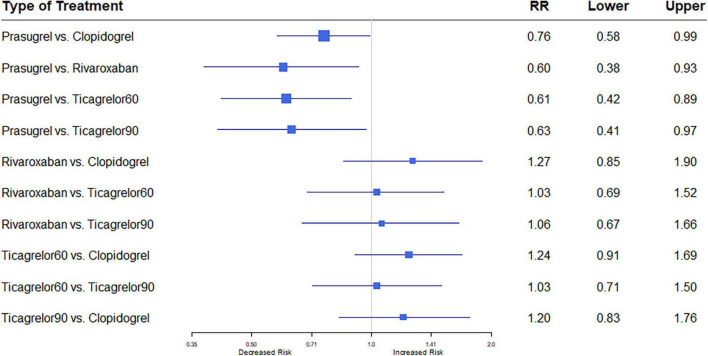
A network meta-analysis of the association between anti-thrombotic therapy and myocardial infarction in patients with chronic coronary syndrome. Net-work Meta-analysis of the Association Between anti-thrombotic therapy and MI. The size of squares is proportional to the weight of each study. Horizontal lines indicate the 95% CI of each treatment; diamond, the pooled estimate with 95% CI; and RR, relative risk.

All eleven trials (7, 8, 12–20) reported the outcome of MI. Among 88,462 patients, 2,823 (3.19%) developed MI. Heterogeneity between the studies when considering MI was *I*^2^ = 37%. Prasugrel plus aspirin was associated with a significantly reduced risk of MI compared to all the dual anti-thrombotic regimens: clopidogrel plus aspirin (RR, 0.76; 95% CI, 0.58–0.99), rivaroxaban plus aspirin (RR, 0.60; 95% CI, 0.38–0.93), ticagrelor 60 plus aspirin (RR, 0.61; 95% CI, 0.42–0.89), and ticagrelor 90 plus aspirin (RR, 0.63; 95% CI, 0.41–0.97) (Figure 4).

#### 3.2.4. Stroke

**FIGURE 5:**
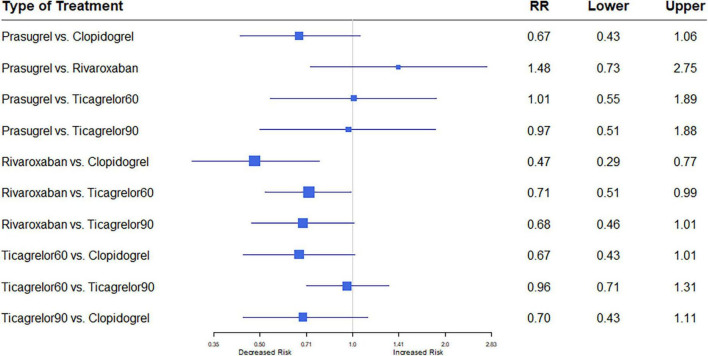
A network meta-analysis of the association between anti-thrombotic therapy and stroke in patients with chronic coronary syndrome. Net-work Meta-analysis of the Association Between anti-thrombotic therapy and stroke. The size of squares is proportional to the weight of each study. Horizontal lines indicate the 95% CI of each treatment; diamond, the pooled estimate with 95% CI; and RR, relative risk.

Nine studies (7, 12–18, 20) reported the outcome of stroke. Among 76,310 patients, 1,098 (1.44%) developed stroke. Heterogeneity between the studies when considering stroke was *I*^2^ = 0%. Rivaroxaban plus aspirin was associated with a significantly reduced risk of stroke compared to clopidogrel plus aspirin (RR, 0.47; 95% CI, 0.29–0.77). Rivaroxaban plus aspirin was also associated with a significantly reduced risk of stroke compared to the low dose ticagrelor plus aspirin regimen (RR, 0.71; 95% CI, 0.51–0.99), whereas compared to the higher dose ticagrelor regimen, a trend was observed (RR, 0.68; 95% CI, 0.46–1.01) (Figure 5).

#### 3.2.5. Stent thrombosis

**FIGURE 6:**
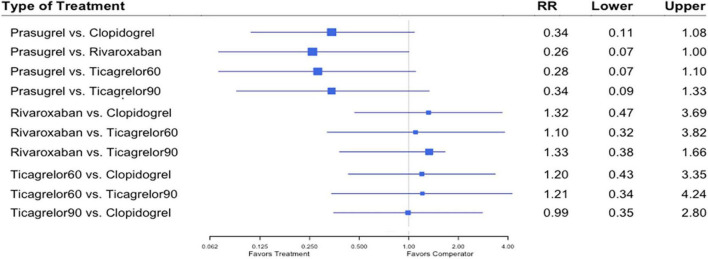
A network meta-analysis of the association between anti-thrombotic therapy and stent thrombosis in patients with chronic coronary syndrome and prior percutaneous coronary intervention. Net-work Meta-analysis of the Association Between anti-thrombotic therapy and stent thrombosis. The size of squares is proportional to the weight of each study. Horizontal lines indicate the 95% CI of each treatment; diamond, the pooled estimate with 95% CI; and RR, relative risk.

Eight studies (7, 8, 16–18, 20–22) reported this outcome. Stent thrombosis events occurred in 349 (0.71%) of 48,825 patients. Heterogeneity between the studies when considering stent thrombosis was *I*^2^ = 37.1%. A trend was observed of lower risk of stent thrombosis in patients treated with the prasugrel compared to the rivaroxaban regimen (RR, 0.26; 95% CI, 0.07–1.00) (Figure 6).

### 3.3. Safety outcomes

#### 3.3.1. Major bleeding

**FIGURE 7:**
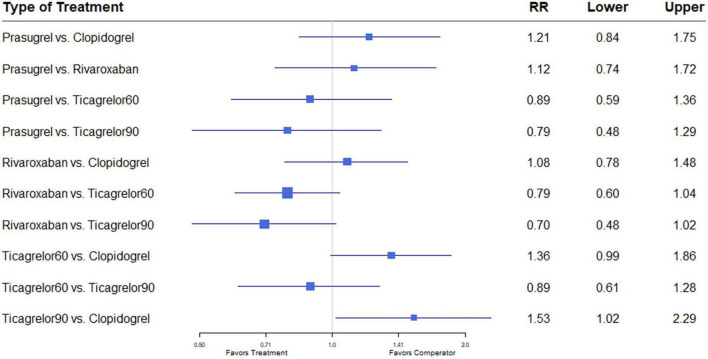
A network meta-analysis of the association between anti-thrombotic therapy and major bleeding in patients with chronic coronary syndrome. Net-work Meta-analysis of the Association Between anti-thrombotic therapy and major bleeding.. The size of squares is proportional to the weight of each study. Horizontal lines indicate the 95% CI of each treatment; diamond, the pooled estimate with 95% CI; and RR, relative risk.

Nine studies (7, 8, 12–18) including 84,851 patients, reported 1,376 (1.62%) major bleeding events, according to the definitions of the individual trials (Supplementary Table 2). Heterogeneity between the studies when considering major bleeding was *I*^2^ = 0%. The results of the NMA showed that compared to the clopidogrel regimen, the incidence of bleeding events was greater with a high dose of ticagrelor (RR, 1.53; 95% CI, 1.02–2.29) (Figure 7).

#### 3.3.2. Intracranial hemorrhage

**FIGURE 8:**
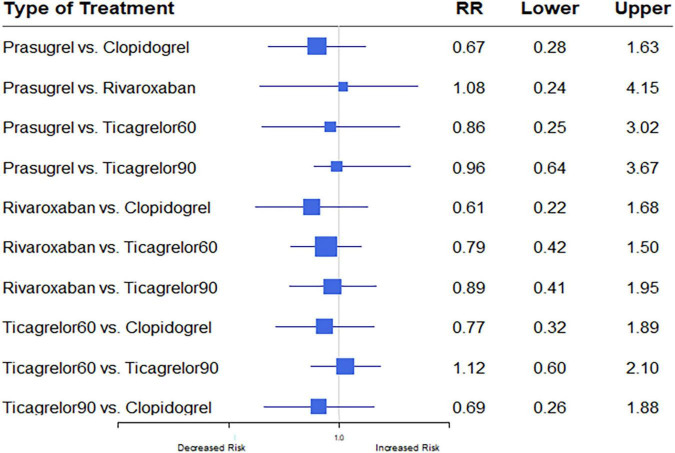
A network meta-analysis of the association between anti-thrombotic therapy and intracerebral hemorrhage in patients with chronic coronary syndrome. The sizes of the squares are proportional to the weights of each study. The horizontal lines indicate the 95% confidence interval (CI) of each treatment. Net-work Meta-analysis of the Association Between anti-thrombotic therapy and ICH. The size of squares is proportional to the weight of each study. Horizontal lines indicate the 95% CI of each treatment; diamond, the pooled estimate with 95% CI; and RR, relative risk.

Eight studies (7, 12–17, 20) reported ICH outcomes. ICH events developed in 291 (0.40%) of 73,199. Heterogeneity between studies when considering ICH was *I*^2^ = 0%. Differences in ICH risk were not statistically significantly between any of the anti-thrombotic strategies investigated (Figure 8).

#### 3.3.3. Ranking of treatment strategies

Table 2 shows the P-score for efficacy and safety outcomes. The rivaroxaban plus aspirin regimen achieved the best performance rankings for all-cause mortality (P-score, 0.969), MACE (P-score, 0.949), and stroke (P-score, 0.960). The worst regimen was clopidogrel plus aspirin for those outcomes, P-scores: 0.210, 0.310, and 0.065, respectively. The best regimen for preventing MI was the prasugrel plus aspirin regimen (P-score, 0.989) followed, in decreasing order, by the regimens that included clopidogrel (P-score, 0.730), ticagrelor 90 (P-score, 0.449), ticagrelor 60 (P-score, 0.405), and rivaroxaban (P-score, 0.360). The same ranking was obtained for the stent thrombosis outcome. Clopidogrel plus aspirin was ranked as the safest regimen for the major bleeding outcome (P-score, 0.694), followed by the rivaroxaban plus aspirin regimen (P-score, 0.591) and the prasugrel plus aspirin regimen (P-score, 0.396). The ticagrelor 60 and 90 plus aspirin regimens appeared as the regimens with the worst performance rankings, P-scores 0.224 and 0.097, respectively. Consistent P-score results were obtained in subgroup analysis evaluating double blinded RCTs for MI, stent thrombosis, and major bleeding outcomes. Due to the lack of data, no comparative analysis was obtained regarding the remaining outcomes (Supplementary Table 6).

**TABLE 2:** P-Scores for the treatment regimens and outcomes.

Treatment regimen	P-Score ranking^a^						
	All-cause mortality	MACEs	MI	Stroke	Stent thrombosis	Major bleeding	ICH
Rivaroxaban	0.969	0.949	0.360	0.960	0.317	0.591	0.621
Prasugrel	0.369	0.486	0.989	0.575	0.968	0.396	0.550
Ticagrelor60	0.632	0.648	0.405	0.622	0.382	0.224	0.347
Ticagrelor90	0.416	0.581	0.449	0.554	0.515	0.097	0.496
Clopidogrel	0.210	0.130	0.730	0.065	0.541	0.694	0.187

P-Score ranking of efficacy and safety outcomes of various dual anti-thrombotic regimens for chronic coronary syndrome.

^a^The P-score represents the probability that each intervention is better than all the competing interventions, as derived from network point estimates and standard errors. MACE, major adverse cardiovascular events; MI, myocardial infarction; ICH, intracerebral hemorrhage.

#### 3.3.4. Quality of studies

Each RCT was assessed for bias (Supplementary Figures 2, 3). The overall risk of bias among the included trials was considered low. All the studies were deemed to have a low risk of bias for random sequence allocation (11/11, 100%) and selective reporting (11/11, 100%); the majority of the studies had low risks for allocation concealment (10/11, 90%), blinding of outcome assessment (8/11, 72%), and incomplete outcome data (9/11, 81%). A few studies were in the highest categories for risk of bias, regarding blinding of participants and personnel (4/11, 36%), and blinding of outcome assessment (2/11, 18%). Unclear risk was the category that included the greatest number of studies with bias (5/11, 45%). The open-label design of six trials (7, 16–20) contributed to the isolated high-risk score for performance bias. Visual inspection of the funnel plot did not reveal any indication of publication bias. The Egger test indicated no statistical significance in asymmetry (Supplementary Figure 4). No source of inconsistency was identified in this NMA for all the outcomes, all the P values were greater than.05.

## 4. Discussion

The current NMA of 11 randomized controlled trials, with 88,462 patients in total, is the first and most comprehensive meta-analysis to evaluate and compare the efficacy and safety of optimal long-term anti-thrombotic treatment for CCS. The anti-thrombotic strategies investigated were the P2Y_12_ inhibitors, namely, clopidogrel, prasugrel, ticagrelor 60/90, and low dose rivaroxaban, in conjunction with aspirin. We report a greater reduction in all-cause mortality, MACE, and stroke following dual therapy based on low dose rivaroxaban (2.5 mg twice daily) plus aspirin compared to clopidogrel plus aspirin. The rivaroxaban regimen was more effective in reducing the risks of stroke and all-cause mortality than the dual anti-platelet regimens, based on clopidogrel and ticagrelor, without an increased risk of major bleeding. Dual platelet inhibition therapy based on prasugrel plus aspirin provided better MI prevention than other long-term anti-thrombotic strategies. Further, the occurrence of fewer ischemic events with the prasugrel regimen was not at the expense of increased bleeding, as none of the dual strategies were associated with differences in either major bleeding or intracranial hemorrhage compared to the prasugrel regimen. Regarding the remaining outcomes, namely MACE, stroke, and all-cause mortality, the prasugrel long-term regimen was non-inferior to the other extended anti-thrombotic strategies.

Direct oral anticoagulants have been tested in two settings for treating coronary artery disease: ACS in the ATLAS ACS 2–TIMI 51 trial and secondary prevention in the COMPASS trial (23, 24). In the ATLAS ACS 2–TIMI 51 trial, rivaroxaban for a mean of 13 months was tested on a background of single or dual antiplatelet therapy in patients with a recent ACS. Rivaroxaban at a dose of 2.5 mg twice daily resulted in lower rates of MACE and mortality compared to a placebo; however, major bleeding and intracranial hemorrhage were increased, but not fatal bleeding. Of note, the regimen tested was based on triple therapy of anti-coagulation and dual anti-platelet therapy (DAPT) consisting of clopidogrel plus aspirin (23). The COMPASS trial investigated patients with stable atherosclerotic vascular disease who were at high risk of recurrent MACE. The primary outcome, a composite of cardiovascular death, stroke, or myocardial infarction, occurred in fewer patients treated with rivaroxaban plus aspirin than with aspirin alone. However, major bleeding events occurred in a higher proportion of the former than the latter (24). The findings of this trial were consistent with the previous trial and has led to the recommendation to consider low-dose rivaroxaban in patients with CCS or symptomatic peripheral artery disease, in addition to the approved FDA indication for rivaroxaban. This renders the factor Xa inhibitor the first direct oral anticoagulant cleared for this use. Our findings corroborate these recommendations, and may suggest a strategy of rivaroxaban 2.5 mg twice daily plus aspirin, in preference to a dual anti-platelet regimen, for patients with CCS who are at high risk of stroke and mortality.

As discussed above, coronary disease develops from stenotic atherosclerotic lesions, which are associated with reduced blood flow and the formation of plaque (25). Three major pathways that amplify platelet activation include the COX-1 pathway, the ADP-P2Y_12_ pathway, and the thrombin pathway (26). Thus, the coagulation-mediated pathway is central to the development of atherosclerosis.

Mechanisms underlying the potential vascular protective effects of rivaroxaban have been described (27). Factor Xa was shown to contribute to atherosclerosis either directly via activation of protease-activated receptors (PARs 1 or 2), or indirectly through the generation of thrombin, which is considered another activator of these receptors. Therefore, rivaroxaban, as a direct factor Xa, inhibited both coagulation and PAR signaling (27).

Our findings suggest DAPT prolongation with prasugrel and aspirin as the superior strategy for reducing MI risk, without increasing the risk of bleeding. The advantage of this strategy is evident from the ISAR-REACT 5 trial (28) and its pharmacodynamic analysis (29). First, platelet-mediated thrombosis is a major pathophysiologic mechanism underlying coronary thrombosis. Therefore, inhibition of platelet activation or aggregation is effective in preventing coronary thrombosis. This is reflected in the superiority of the dual anti-platelet compared to the anti-coagulation regimen in the reduction of MI and stent thrombosis, and can explain the inferiority of the rivaroxaban-based regimen regarding these outcomes, and the consequent requirement for anti-platelet therapy. Second, several studies have shown that prasugrel compared to ticagrelor was associated with stronger platelet inhibition (29–31), improved endothelial function, and reduced IL-6 levels (30, 31), all of which may have predictive implications.

In addition to the above, pharmacodynamic analysis of the ISAR-REACT 5 trial demonstrated lower levels of adenosine diphosphate–induced platelet aggregation in patients treated with prasugrel compared to ticagrelor. Moreover, the incidence of the primary endpoint increased across a certain percentage of platelet aggregation, independent of the assigned study drug (29). Hence, while both agents are considered potent inhibitors of P2Y_12_ receptor, their mechanisms of action are different. In their explanation of the findings, the authors focused on the issue of compliance. Specifically, the consequences of non-compliance may be greater for patients taking ticagrelor, given that its twice-daily regimen does not irreversibly and competitively inhibit the P2Y_12_ receptor. Notably, a number of studies showed that significantly more patients stopped ticagrelor due to dyspnea adverse events (13, 15, 28, 30). The lower compliance of the ticagrelor regimen and its different mechanism on platelet inhibition may explain its inferiority. Taken together, our findings show consistent efficacy of prasugrel overall, independent of the specific stage of the coronary syndrome, acute or chronic.

We report lower incidence, though without statistical significance, of stent thrombosis among patients treated with prasugrel than according to the other examined strategies. The low number of events, due to the rare occurrence of complications of late stent thrombosis, may explain the lack of statistical significance.

Extending the 2019 ESC guidelines (1) the 2020 NSTEMI ESC guidelines (31) stratified patients to two risk groups. High versus moderately increased risk for thrombosis were categorized as complex or non-complex coronary artery disease, according to clinical judgment and consideration of the patient’s cardiovascular history and coronary anatomy.

Our NMA investigated the situation in which the decision on long-term treatment had already been made. This contrasts with numerous meta-analyses that examined the decision of whether to start a prolonged therapy (32–34). An NMA published in 2021 (35) included four RCTs, and raised the same topic as our NMA. The conclusion was that of all the anti-thrombotic strategies investigated, rivaroxaban plus aspirin resulted in fewer anti-ischemic events, except for MI. This considered the outcomes of all ischemic and bleeding events and all-cause mortality. Hence, rivaroxaban plus aspirin appeared as the preferred long-term anti-thrombotic regimen for patients with CCS and high-risk factors. In that NMA, thienopyridine plus aspirin seemed safer and even more effective than the ticagrelor plus aspirin regimen. Our findings are partially consistent with theirs, though investigation of the non-separated thienopyridine regimens underestimated the treatment effect of each agent. Of note, the DAPT study (8) did not report an analysis of individual outcomes such as stroke or all-cause mortality, stratified by the type of P2Y_12_ inhibitor (clopidogrel or prasugrel). Therefore, the non-significant results regarding MACE as a composite of these individual outcomes for each thienopyridine plus aspirin regimen may be due to the lack of such analyses. Regarding safety outcomes, ticagrelor plus aspirin regimen appeared to show lower safety than the other regimens, which is consistent with our NMA.

In contrast to our methodology, the abovementioned NMA considered the thienopyridine group as a single intervention, despite the investigation of two agents with unequal potency. By not assessing separately the magnitude of efficacy and safety of each agent, the analysis evidently missed their full effects. This applies also to the ticagrelor regimen, when the high and low doses were not considered separated as independent interventions. Moreover, the different definitions between trials, of MACE, the primary efficacy outcome, may have introduced bias.

The current NMA on long-term dual anti-thrombotic therapy for CCS was larger than the previous one. We included 11 trials compared to four trials. Importantly, we considered separately, the type and dosage, of each P2Y_12_ inhibitor. However, in the absence of direct evidence our conclusions are only hypothesis generating and need to establish a clinical multiple drug comparison.

### 5. Limitations

Our NMA has several limitations that need to be considered when interpreting the findings. First, although clear statistical heterogeneity was not observed, the data were gathered from study-level data, and conclusions were drawn from pooled trials. The heterogeneous populations inevitably differed in the severity of disease presentation, the cardiovascular risk profile of the patients, and the follow-up periods; and slightly in definitions of outcomes. These differences may have affected the results. However, a comprehensive systematic review of the literature was conducted; and RCTs were included that assessed long-term anti-thrombotic strategies in stable patients with CCS, or in mixed populations comprising both patients with ACS and CCS. In regard to acute disease, it should be emphasized that an inclusion criterion was that the patients were treated by the investigated agents during at least 12 months, thus indicating that the patients had already reached the chronic stage. A second limitation is that some of the RCTs were unblinded. This may bias the reporting of the outcomes. However, these unblinded trials provided only 16% of the total population studied, subgroup analysis on blinded RCTs was conducted and similar results were obtained. A third limitation is the lack of any sub-analysis for individual outcomes that stratified by the type of P2Y_12_ inhibitor in the DAPT study. Finally, a few trials with each exploratory treatment group were included in this analysis, particularly, data on rivaroxaban treatment group was derived from COMPASS study (14, 22). In fact, these limitations may obscure the complete clinical picture regarding the actual risk and benefits of each intervention, therefore, more studies with direct comparisons are needed in the future to provide more robust results.

## 6. Conclusion

In patients with CCS and high-risk thrombotic factors, rivaroxaban plus aspirin appeared as preferred long-term anti-thrombotic regimen in preventing cerebrovascular events and all-cause mortality, compared with dual therapy, based on clopidogrel or ticagrelor, without increased risk of major bleeding. Our available results tend to support the efficacy of extended anti-thrombotic therapy consisting of prasugrel plus aspirin in lowering MI incidence compared to the other strategies, without an increased risk of bleeding. However, additional large-scale direct clinical trials must be conducted to further determine the adequate long-term anti-thrombotic regimens for patients with CCS.

## Data availability statement

The original contributions presented in this study are included in the article/Supplementary material, further inquiries can be directed to the corresponding author.

## Author contributions

NA, GH, and BR performed the literature search. NA performed the literature screening, data extraction, risk of bias evaluation, drafted the initial manuscript, and wrote the final manuscript. BR, IM, VR, NA, and SY conducted the analyses. All the authors contributed to the study protocol, commented on early versions of the manuscript, and read and approved the submitted version.
