# Supplementary material for: Efficacy and safety outcomes of long-term anti-thrombotic treatment of chronic coronary artery disease: A systematic review and network meta-analysis
Source: Front Cardiovasc Med. 2023 Jan 9;9:1016390. doi: 10.3389/fcvm.2022.1016390 (PMC9868614; doi:10.3389/fcvm.2022.1016390)
Supplement: Supplementary file 1 [file Data_Sheet_1.docx]

‏17/12/2022

Supplements

[Table S1. Syntaxes in Database Searches 2](#_Toc122987369)

[Embase 2](#_Toc122987370)

[PubMed 13](#_Toc122987371)

[Cochrane 35](#_Toc122987372)

[Clinical trials 36](#_Toc122987373)

[Table S2. Outcome event definitions 38](#_Toc122987374)

[Table S3. Clinical Presentation at Enrollment 44](#_Toc122987375)

[Table S4. Medical Therapy Used in Included Trials 45](#_Toc122987376)

[Table S5. Baseline patients’ characteristics 46](#_Toc122987377)

[Table S6 : P-Scores for the treatment regimens and outcomes in subgroup analysis 49](#_Toc122987378)

[Figure S1. Diagram of network meta-analysis 50](#_Toc122987379)

[Figure S2. Risk of bias assessment for randomized control studies, evaluated by the Cochrane Collaboration's Risk of Bias Tool 51](#_Toc122987380)

[Figure S3. Risk of bias graph : review authors' judgements about each risk of bias item presented as percentages across all included studies 52](#_Toc122987381)

[Figure S4. myocardial infarction funnel plot and Egger's regression test 53](#_Toc122987382)

[References 54](#_Toc122987383)

# Table S1. Syntaxes in Database Searches

# Embase

|  | Query | Results |
| --- | --- | --- |
| #38 | (((('drug regimen':ti,ab OR combination:ti,ab OR dual:ti,ab) OR 'drug combination'/exp) AND ('randomized controlled trial'/exp OR 'controlled clinical trial'/exp OR randomized:ti,ab OR placebo:ti,ab OR randomly:ti,ab OR trial:ti) AND ((((antiplatelet:ti,ab OR clopidogrel:ti,ab OR ticlopidine:ti,ab OR plavix:ti,ab OR rivaroxaban:ti,ab OR clopilet:ti,ab OR grepid:ti,ab OR zopya:ti,ab OR zylagren:ti,ab OR zyllt:ti,ab OR cangrelor:ti,ab OR elinogrel:ti,ab OR prasugrel:ti,ab OR regrelor:ti,ab OR selatogrel:ti,ab OR ticagrelor:ti,ab OR effient:ti,ab OR efient:ti,ab OR brilinta:ti,ab OR brilique:ti,ab OR possia:ti,ab OR p2y12:ti,ab OR thienopyridine:ti,ab) OR 'purinergic p2y receptor antagonist'/exp) AND (('acetylsalycic acid':ti,ab OR 'acetylsalycylic acid':ti,ab OR aspirin:ti,ab OR aspirine:ti,ab OR 'acetylsalicylic acid':ti,ab OR acylpyrin:ti,ab OR aloxiprimum:ti,ab OR colfarit:ti,ab OR dispril:ti,ab OR easprin:ti,ab OR ecotrin:ti,ab OR endosprin:ti,ab OR magnecyl:ti,ab OR micristin:ti,ab OR polopirin:ti,ab OR polopiryna:ti,ab OR solprin:ti,ab OR solupsan:ti,ab OR zorprin:ti,ab OR acetysal:ti,ab OR 'cyclooxygenase inhibitor':ti,ab) OR 'acetylsalicylic acid'/exp)) OR ((('acetylsalycic acid':ti,ab OR 'acetylsalycylic acid':ti,ab OR aspirin:ti,ab OR aspirine:ti,ab OR 'acetylsalicylic acid':ti,ab OR acylpyrin:ti,ab OR aloxiprimum:ti,ab OR colfarit:ti,ab OR dispril:ti,ab OR easprin:ti,ab OR ecotrin:ti,ab OR endosprin:ti,ab OR magnecyl:ti,ab OR micristin:ti,ab OR polopirin:ti,ab OR polopiryna:ti,ab OR solprin:ti,ab OR solupsan:ti,ab OR zorprin:ti,ab OR acetysal:ti,ab OR 'cyclooxygenase inhibitor':ti,ab) OR 'acetylsalicylic acid'/exp) AND ((anticoagul*:ti,ab OR antithrombin:ti,ab OR 'factor xa inhibitor':ti,ab OR xarelto:ti,ab OR rivaroxaban:ti,ab OR apixaban:ti,ab OR pradaxa:ti,ab OR eliquis:ti,ab OR dabigatran:ti,ab OR noac:ti,ab OR doac:ti,ab) OR 'hematologic agent'/exp))) AND ((('cardiovascular disease':ti,ab OR 'vascular disease':ti,ab OR 'heart disease':ti,ab OR 'cardiac disorder':ti,ab OR 'heart disorder':ti,ab OR coronary:ti,ab OR angina:ti,ab OR 'peripheral disease':ti,ab OR 'myocardial reperfusion':ti,ab OR 'arterial obstructive diseases':ti,ab OR 'peripheral angiopathy':ti,ab OR 'major adverse cardiovascular event':ti,ab OR cardiopathy:ti,ab OR angiopathy:ti,ab OR 'blood vessel disease':ti,ab OR vasculopathy:ti,ab OR 'heart attack':ti,ab OR 'cardiovascular stroke':ti,ab OR atherosclero*:ti,ab OR isch*:ti,ab OR infarction:ti,ab OR stemi:ti,ab OR thrombo*:ti,ab OR stable:ti,ab) OR 'cardiovascular disease'/exp) OR (('percutaneous coronary intervention':ti,ab OR 'percutaneous coronary revascularizations':ti,ab OR 'percutaneous transluminal angioplasty':ti,ab) OR 'interventional cardiovascular procedure'/exp) OR (('drug eluting cardiovascular stent':ti,ab OR 'drug eluting coronary artery stent':ti,ab OR 'drug eluting stent':ti,ab) OR 'drug eluting stent'/exp)) AND ((('chronic treatment':ti,ab OR 'life support care':ti,ab OR 'long term':ti,ab) OR 'long term care'/exp) OR ((duration:ti,ab OR length:ti,ab OR extended:ti,ab OR continue:ti,ab OR follow:ti,ab OR beyond:ti,ab) OR 'treatment duration'/exp))) NOT 'review'/exp) AND 'article'/exp | 1106 |
| #37 | 'article'/exp | 21539589 |
| #36 | ((('drug regimen':ti,ab OR combination:ti,ab OR dual:ti,ab) OR 'drug combination'/exp) AND ('randomized controlled trial'/exp OR 'controlled clinical trial'/exp OR randomized:ti,ab OR placebo:ti,ab OR randomly:ti,ab OR trial:ti) AND ((((antiplatelet:ti,ab OR clopidogrel:ti,ab OR ticlopidine:ti,ab OR plavix:ti,ab OR rivaroxaban:ti,ab OR clopilet:ti,ab OR grepid:ti,ab OR zopya:ti,ab OR zylagren:ti,ab OR zyllt:ti,ab OR cangrelor:ti,ab OR elinogrel:ti,ab OR prasugrel:ti,ab OR regrelor:ti,ab OR selatogrel:ti,ab OR ticagrelor:ti,ab OR effient:ti,ab OR efient:ti,ab OR brilinta:ti,ab OR brilique:ti,ab OR possia:ti,ab OR p2y12:ti,ab OR thienopyridine:ti,ab) OR 'purinergic p2y receptor antagonist'/exp) AND (('acetylsalycic acid':ti,ab OR 'acetylsalycylic acid':ti,ab OR aspirin:ti,ab OR aspirine:ti,ab OR 'acetylsalicylic acid':ti,ab OR acylpyrin:ti,ab OR aloxiprimum:ti,ab OR colfarit:ti,ab OR dispril:ti,ab OR easprin:ti,ab OR ecotrin:ti,ab OR endosprin:ti,ab OR magnecyl:ti,ab OR micristin:ti,ab OR polopirin:ti,ab OR polopiryna:ti,ab OR solprin:ti,ab OR solupsan:ti,ab OR zorprin:ti,ab OR acetysal:ti,ab OR 'cyclooxygenase inhibitor':ti,ab) OR 'acetylsalicylic acid'/exp)) OR ((('acetylsalycic acid':ti,ab OR 'acetylsalycylic acid':ti,ab OR aspirin:ti,ab OR aspirine:ti,ab OR 'acetylsalicylic acid':ti,ab OR acylpyrin:ti,ab OR aloxiprimum:ti,ab OR colfarit:ti,ab OR dispril:ti,ab OR easprin:ti,ab OR ecotrin:ti,ab OR endosprin:ti,ab OR magnecyl:ti,ab OR micristin:ti,ab OR polopirin:ti,ab OR polopiryna:ti,ab OR solprin:ti,ab OR solupsan:ti,ab OR zorprin:ti,ab OR acetysal:ti,ab OR 'cyclooxygenase inhibitor':ti,ab) OR 'acetylsalicylic acid'/exp) AND ((anticoagul*:ti,ab OR antithrombin:ti,ab OR 'factor xa inhibitor':ti,ab OR xarelto:ti,ab OR rivaroxaban:ti,ab OR apixaban:ti,ab OR pradaxa:ti,ab OR eliquis:ti,ab OR dabigatran:ti,ab OR noac:ti,ab OR doac:ti,ab) OR 'hematologic agent'/exp))) AND ((('cardiovascular disease':ti,ab OR 'vascular disease':ti,ab OR 'heart disease':ti,ab OR 'cardiac disorder':ti,ab OR 'heart disorder':ti,ab OR coronary:ti,ab OR angina:ti,ab OR 'peripheral disease':ti,ab OR 'myocardial reperfusion':ti,ab OR 'arterial obstructive diseases':ti,ab OR 'peripheral angiopathy':ti,ab OR 'major adverse cardiovascular event':ti,ab OR cardiopathy:ti,ab OR angiopathy:ti,ab OR 'blood vessel disease':ti,ab OR vasculopathy:ti,ab OR 'heart attack':ti,ab OR 'cardiovascular stroke':ti,ab OR atherosclero*:ti,ab OR isch*:ti,ab OR infarction:ti,ab OR stemi:ti,ab OR thrombo*:ti,ab OR stable:ti,ab) OR 'cardiovascular disease'/exp) OR (('percutaneous coronary intervention':ti,ab OR 'percutaneous coronary revascularizations':ti,ab OR 'percutaneous transluminal angioplasty':ti,ab) OR 'interventional cardiovascular procedure'/exp) OR (('drug eluting cardiovascular stent':ti,ab OR 'drug eluting coronary artery stent':ti,ab OR 'drug eluting stent':ti,ab) OR 'drug eluting stent'/exp)) AND ((('chronic treatment':ti,ab OR 'life support care':ti,ab OR 'long term':ti,ab) OR 'long term care'/exp) OR ((duration:ti,ab OR length:ti,ab OR extended:ti,ab OR continue:ti,ab OR follow:ti,ab OR beyond:ti,ab) OR 'treatment duration'/exp))) NOT 'review'/exp | 1596 |
| #35 | 'review'/exp | 2641044 |
| #34 | (('drug regimen':ti,ab OR combination:ti,ab OR dual:ti,ab) OR 'drug combination'/exp) AND ('randomized controlled trial'/exp OR 'controlled clinical trial'/exp OR randomized:ti,ab OR placebo:ti,ab OR randomly:ti,ab OR trial:ti) AND ((((antiplatelet:ti,ab OR clopidogrel:ti,ab OR ticlopidine:ti,ab OR plavix:ti,ab OR rivaroxaban:ti,ab OR clopilet:ti,ab OR grepid:ti,ab OR zopya:ti,ab OR zylagren:ti,ab OR zyllt:ti,ab OR cangrelor:ti,ab OR elinogrel:ti,ab OR prasugrel:ti,ab OR regrelor:ti,ab OR selatogrel:ti,ab OR ticagrelor:ti,ab OR effient:ti,ab OR efient:ti,ab OR brilinta:ti,ab OR brilique:ti,ab OR possia:ti,ab OR p2y12:ti,ab OR thienopyridine:ti,ab) OR 'purinergic p2y receptor antagonist'/exp) AND (('acetylsalycic acid':ti,ab OR 'acetylsalycylic acid':ti,ab OR aspirin:ti,ab OR aspirine:ti,ab OR 'acetylsalicylic acid':ti,ab OR acylpyrin:ti,ab OR aloxiprimum:ti,ab OR colfarit:ti,ab OR dispril:ti,ab OR easprin:ti,ab OR ecotrin:ti,ab OR endosprin:ti,ab OR magnecyl:ti,ab OR micristin:ti,ab OR polopirin:ti,ab OR polopiryna:ti,ab OR solprin:ti,ab OR solupsan:ti,ab OR zorprin:ti,ab OR acetysal:ti,ab OR 'cyclooxygenase inhibitor':ti,ab) OR 'acetylsalicylic acid'/exp)) OR ((('acetylsalycic acid':ti,ab OR 'acetylsalycylic acid':ti,ab OR aspirin:ti,ab OR aspirine:ti,ab OR 'acetylsalicylic acid':ti,ab OR acylpyrin:ti,ab OR aloxiprimum:ti,ab OR colfarit:ti,ab OR dispril:ti,ab OR easprin:ti,ab OR ecotrin:ti,ab OR endosprin:ti,ab OR magnecyl:ti,ab OR micristin:ti,ab OR polopirin:ti,ab OR polopiryna:ti,ab OR solprin:ti,ab OR solupsan:ti,ab OR zorprin:ti,ab OR acetysal:ti,ab OR 'cyclooxygenase inhibitor':ti,ab) OR 'acetylsalicylic acid'/exp) AND ((anticoagul*:ti,ab OR antithrombin:ti,ab OR 'factor xa inhibitor':ti,ab OR xarelto:ti,ab OR rivaroxaban:ti,ab OR apixaban:ti,ab OR pradaxa:ti,ab OR eliquis:ti,ab OR dabigatran:ti,ab OR noac:ti,ab OR doac:ti,ab) OR 'hematologic agent'/exp))) AND ((('cardiovascular disease':ti,ab OR 'vascular disease':ti,ab OR 'heart disease':ti,ab OR 'cardiac disorder':ti,ab OR 'heart disorder':ti,ab OR coronary:ti,ab OR angina:ti,ab OR 'peripheral disease':ti,ab OR 'myocardial reperfusion':ti,ab OR 'arterial obstructive diseases':ti,ab OR 'peripheral angiopathy':ti,ab OR 'major adverse cardiovascular event':ti,ab OR cardiopathy:ti,ab OR angiopathy:ti,ab OR 'blood vessel disease':ti,ab OR vasculopathy:ti,ab OR 'heart attack':ti,ab OR 'cardiovascular stroke':ti,ab OR atherosclero*:ti,ab OR isch*:ti,ab OR infarction:ti,ab OR stemi:ti,ab OR thrombo*:ti,ab OR stable:ti,ab) OR 'cardiovascular disease'/exp) OR (('percutaneous coronary intervention':ti,ab OR 'percutaneous coronary revascularizations':ti,ab OR 'percutaneous transluminal angioplasty':ti,ab) OR 'interventional cardiovascular procedure'/exp) OR (('drug eluting cardiovascular stent':ti,ab OR 'drug eluting coronary artery stent':ti,ab OR 'drug eluting stent':ti,ab) OR 'drug eluting stent'/exp)) AND ((('chronic treatment':ti,ab OR 'life support care':ti,ab OR 'long term':ti,ab) OR 'long term care'/exp) OR ((duration:ti,ab OR length:ti,ab OR extended:ti,ab OR continue:ti,ab OR follow:ti,ab OR beyond:ti,ab) OR 'treatment duration'/exp)) | 2013 |
| #33 | (('chronic treatment':ti,ab OR 'life support care':ti,ab OR 'long term':ti,ab) OR 'long term care'/exp) OR ((duration:ti,ab OR length:ti,ab OR extended:ti,ab OR continue:ti,ab OR follow:ti,ab OR beyond:ti,ab) OR 'treatment duration'/exp) | 4983758 |
| #32 | (('cardiovascular disease':ti,ab OR 'vascular disease':ti,ab OR 'heart disease':ti,ab OR 'cardiac disorder':ti,ab OR 'heart disorder':ti,ab OR coronary:ti,ab OR angina:ti,ab OR 'peripheral disease':ti,ab OR 'myocardial reperfusion':ti,ab OR 'arterial obstructive diseases':ti,ab OR 'peripheral angiopathy':ti,ab OR 'major adverse cardiovascular event':ti,ab OR cardiopathy:ti,ab OR angiopathy:ti,ab OR 'blood vessel disease':ti,ab OR vasculopathy:ti,ab OR 'heart attack':ti,ab OR 'cardiovascular stroke':ti,ab OR atherosclero*:ti,ab OR isch*:ti,ab OR infarction:ti,ab OR stemi:ti,ab OR thrombo*:ti,ab OR stable:ti,ab) OR 'cardiovascular disease'/exp) OR (('percutaneous coronary intervention':ti,ab OR 'percutaneous coronary revascularizations':ti,ab OR 'percutaneous transluminal angioplasty':ti,ab) OR 'interventional cardiovascular procedure'/exp) OR (('drug eluting cardiovascular stent':ti,ab OR 'drug eluting coronary artery stent':ti,ab OR 'drug eluting stent':ti,ab) OR 'drug eluting stent'/exp) | 5330601 |
| #31 | (((antiplatelet:ti,ab OR clopidogrel:ti,ab OR ticlopidine:ti,ab OR plavix:ti,ab OR rivaroxaban:ti,ab OR clopilet:ti,ab OR grepid:ti,ab OR zopya:ti,ab OR zylagren:ti,ab OR zyllt:ti,ab OR cangrelor:ti,ab OR elinogrel:ti,ab OR prasugrel:ti,ab OR regrelor:ti,ab OR selatogrel:ti,ab OR ticagrelor:ti,ab OR effient:ti,ab OR efient:ti,ab OR brilinta:ti,ab OR brilique:ti,ab OR possia:ti,ab OR p2y12:ti,ab OR thienopyridine:ti,ab) OR 'purinergic p2y receptor antagonist'/exp) AND (('acetylsalycic acid':ti,ab OR 'acetylsalycylic acid':ti,ab OR aspirin:ti,ab OR aspirine:ti,ab OR 'acetylsalicylic acid':ti,ab OR acylpyrin:ti,ab OR aloxiprimum:ti,ab OR colfarit:ti,ab OR dispril:ti,ab OR easprin:ti,ab OR ecotrin:ti,ab OR endosprin:ti,ab OR magnecyl:ti,ab OR micristin:ti,ab OR polopirin:ti,ab OR polopiryna:ti,ab OR solprin:ti,ab OR solupsan:ti,ab OR zorprin:ti,ab OR acetysal:ti,ab OR 'cyclooxygenase inhibitor':ti,ab) OR 'acetylsalicylic acid'/exp)) OR ((('acetylsalycic acid':ti,ab OR 'acetylsalycylic acid':ti,ab OR aspirin:ti,ab OR aspirine:ti,ab OR 'acetylsalicylic acid':ti,ab OR acylpyrin:ti,ab OR aloxiprimum:ti,ab OR colfarit:ti,ab OR dispril:ti,ab OR easprin:ti,ab OR ecotrin:ti,ab OR endosprin:ti,ab OR magnecyl:ti,ab OR micristin:ti,ab OR polopirin:ti,ab OR polopiryna:ti,ab OR solprin:ti,ab OR solupsan:ti,ab OR zorprin:ti,ab OR acetysal:ti,ab OR 'cyclooxygenase inhibitor':ti,ab) OR 'acetylsalicylic acid'/exp) AND ((anticoagul*:ti,ab OR antithrombin:ti,ab OR 'factor xa inhibitor':ti,ab OR xarelto:ti,ab OR rivaroxaban:ti,ab OR apixaban:ti,ab OR pradaxa:ti,ab OR eliquis:ti,ab OR dabigatran:ti,ab OR noac:ti,ab OR doac:ti,ab) OR 'hematologic agent'/exp)) | 215912 |
| #30 | (('acetylsalycic acid':ti,ab OR 'acetylsalycylic acid':ti,ab OR aspirin:ti,ab OR aspirine:ti,ab OR 'acetylsalicylic acid':ti,ab OR acylpyrin:ti,ab OR aloxiprimum:ti,ab OR colfarit:ti,ab OR dispril:ti,ab OR easprin:ti,ab OR ecotrin:ti,ab OR endosprin:ti,ab OR magnecyl:ti,ab OR micristin:ti,ab OR polopirin:ti,ab OR polopiryna:ti,ab OR solprin:ti,ab OR solupsan:ti,ab OR zorprin:ti,ab OR acetysal:ti,ab OR 'cyclooxygenase inhibitor':ti,ab) OR 'acetylsalicylic acid'/exp) AND ((anticoagul*:ti,ab OR antithrombin:ti,ab OR 'factor xa inhibitor':ti,ab OR xarelto:ti,ab OR rivaroxaban:ti,ab OR apixaban:ti,ab OR pradaxa:ti,ab OR eliquis:ti,ab OR dabigatran:ti,ab OR noac:ti,ab OR doac:ti,ab) OR 'hematologic agent'/exp) | 215582 |
| #29 | ((antiplatelet:ti,ab OR clopidogrel:ti,ab OR ticlopidine:ti,ab OR plavix:ti,ab OR rivaroxaban:ti,ab OR clopilet:ti,ab OR grepid:ti,ab OR zopya:ti,ab OR zylagren:ti,ab OR zyllt:ti,ab OR cangrelor:ti,ab OR elinogrel:ti,ab OR prasugrel:ti,ab OR regrelor:ti,ab OR selatogrel:ti,ab OR ticagrelor:ti,ab OR effient:ti,ab OR efient:ti,ab OR brilinta:ti,ab OR brilique:ti,ab OR possia:ti,ab OR p2y12:ti,ab OR thienopyridine:ti,ab) OR 'purinergic p2y receptor antagonist'/exp) AND (('acetylsalycic acid':ti,ab OR 'acetylsalycylic acid':ti,ab OR aspirin:ti,ab OR aspirine:ti,ab OR 'acetylsalicylic acid':ti,ab OR acylpyrin:ti,ab OR aloxiprimum:ti,ab OR colfarit:ti,ab OR dispril:ti,ab OR easprin:ti,ab OR ecotrin:ti,ab OR endosprin:ti,ab OR magnecyl:ti,ab OR micristin:ti,ab OR polopirin:ti,ab OR polopiryna:ti,ab OR solprin:ti,ab OR solupsan:ti,ab OR zorprin:ti,ab OR acetysal:ti,ab OR 'cyclooxygenase inhibitor':ti,ab) OR 'acetylsalicylic acid'/exp) | 58037 |
| #28 | 'randomized controlled trial'/exp OR 'controlled clinical trial'/exp OR randomized:ti,ab OR placebo:ti,ab OR randomly:ti,ab OR trial:ti | 1613489 |
| #27 | (duration:ti,ab OR length:ti,ab OR extended:ti,ab OR continue:ti,ab OR follow:ti,ab OR beyond:ti,ab) OR 'treatment duration'/exp | 3770195 |
| #26 | 'treatment duration'/exp | 225991 |
| #25 | duration:ti,ab OR length:ti,ab OR extended:ti,ab OR continue:ti,ab OR follow:ti,ab OR beyond:ti,ab | 3630669 |
| #24 | ('chronic treatment':ti,ab OR 'life support care':ti,ab OR 'long term':ti,ab) OR 'long term care'/exp | 2537130 |
| #23 | 'long term care'/exp | 1748413 |
| #22 | 'chronic treatment':ti,ab OR 'life support care':ti,ab OR 'long term':ti,ab | 1093873 |
| #21 | ('drug eluting cardiovascular stent':ti,ab OR 'drug eluting coronary artery stent':ti,ab OR 'drug eluting stent':ti,ab) OR 'drug eluting stent'/exp | 33658 |
| #20 | 'drug eluting stent'/exp | 33223 |
| #19 | 'drug eluting cardiovascular stent':ti,ab OR 'drug eluting coronary artery stent':ti,ab OR 'drug eluting stent':ti,ab | 9050 |
| #18 | ('percutaneous coronary intervention':ti,ab OR 'percutaneous coronary revascularizations':ti,ab OR 'percutaneous transluminal angioplasty':ti,ab) OR 'interventional cardiovascular procedure'/exp | 147497 |
| #17 | 'interventional cardiovascular procedure'/exp | 142990 |
| #16 | 'percutaneous coronary intervention':ti,ab OR 'percutaneous coronary revascularizations':ti,ab OR 'percutaneous transluminal angioplasty':ti,ab | 57852 |
| #15 | ('cardiovascular disease':ti,ab OR 'vascular disease':ti,ab OR 'heart disease':ti,ab OR 'cardiac disorder':ti,ab OR 'heart disorder':ti,ab OR coronary:ti,ab OR angina:ti,ab OR 'peripheral disease':ti,ab OR 'myocardial reperfusion':ti,ab OR 'arterial obstructive diseases':ti,ab OR 'peripheral angiopathy':ti,ab OR 'major adverse cardiovascular event':ti,ab OR cardiopathy:ti,ab OR angiopathy:ti,ab OR 'blood vessel disease':ti,ab OR vasculopathy:ti,ab OR 'heart attack':ti,ab OR 'cardiovascular stroke':ti,ab OR atherosclero*:ti,ab OR isch*:ti,ab OR infarction:ti,ab OR stemi:ti,ab OR thrombo*:ti,ab OR stable:ti,ab) OR 'cardiovascular disease'/exp | 5324664 |
| #14 | 'cardiovascular disease'/exp | 4366554 |
| #13 | 'cardiovascular disease':ti,ab OR 'vascular disease':ti,ab OR 'heart disease':ti,ab OR 'cardiac disorder':ti,ab OR 'heart disorder':ti,ab OR coronary:ti,ab OR angina:ti,ab OR 'peripheral disease':ti,ab OR 'myocardial reperfusion':ti,ab OR 'arterial obstructive diseases':ti,ab OR 'peripheral angiopathy':ti,ab OR 'major adverse cardiovascular event':ti,ab OR cardiopathy:ti,ab OR angiopathy:ti,ab OR 'blood vessel disease':ti,ab OR vasculopathy:ti,ab OR 'heart attack':ti,ab OR 'cardiovascular stroke':ti,ab OR atherosclero*:ti,ab OR isch*:ti,ab OR infarction:ti,ab OR stemi:ti,ab OR thrombo*:ti,ab OR stable:ti,ab | 2586472 |
| #12 | ('drug regimen':ti,ab OR combination:ti,ab OR dual:ti,ab) OR 'drug combination'/exp | 1409221 |
| #11 | 'drug combination'/exp | 151995 |
| #10 | 'drug regimen':ti,ab OR combination:ti,ab OR dual:ti,ab | 1317293 |
| #9 | (anticoagul*:ti,ab OR antithrombin:ti,ab OR 'factor xa inhibitor':ti,ab OR xarelto:ti,ab OR rivaroxaban:ti,ab OR apixaban:ti,ab OR pradaxa:ti,ab OR eliquis:ti,ab OR dabigatran:ti,ab OR noac:ti,ab OR doac:ti,ab) OR 'hematologic agent'/exp | 1410403 |
| #8 | 'hematologic agent'/exp | 1379595 |
| #7 | anticoagul*:ti,ab OR antithrombin:ti,ab OR 'factor xa inhibitor':ti,ab OR xarelto:ti,ab OR rivaroxaban:ti,ab OR apixaban:ti,ab OR pradaxa:ti,ab OR eliquis:ti,ab OR dabigatran:ti,ab OR noac:ti,ab OR doac:ti,ab | 165546 |
| #6 | ('acetylsalycic acid':ti,ab OR 'acetylsalycylic acid':ti,ab OR aspirin:ti,ab OR aspirine:ti,ab OR 'acetylsalicylic acid':ti,ab OR acylpyrin:ti,ab OR aloxiprimum:ti,ab OR colfarit:ti,ab OR dispril:ti,ab OR easprin:ti,ab OR ecotrin:ti,ab OR endosprin:ti,ab OR magnecyl:ti,ab OR micristin:ti,ab OR polopirin:ti,ab OR polopiryna:ti,ab OR solprin:ti,ab OR solupsan:ti,ab OR zorprin:ti,ab OR acetysal:ti,ab OR 'cyclooxygenase inhibitor':ti,ab) OR 'acetylsalicylic acid'/exp | 224590 |
| #5 | 'acetylsalicylic acid'/exp | 211914 |
| #4 | 'acetylsalycic acid':ti,ab OR 'acetylsalycylic acid':ti,ab OR aspirin:ti,ab OR aspirine:ti,ab OR 'acetylsalicylic acid':ti,ab OR acylpyrin:ti,ab OR aloxiprimum:ti,ab OR colfarit:ti,ab OR dispril:ti,ab OR easprin:ti,ab OR ecotrin:ti,ab OR endosprin:ti,ab OR magnecyl:ti,ab OR micristin:ti,ab OR polopirin:ti,ab OR polopiryna:ti,ab OR solprin:ti,ab OR solupsan:ti,ab OR zorprin:ti,ab OR acetysal:ti,ab OR 'cyclooxygenase inhibitor':ti,ab | 87236 |
| #3 | (antiplatelet:ti,ab OR clopidogrel:ti,ab OR ticlopidine:ti,ab OR plavix:ti,ab OR rivaroxaban:ti,ab OR clopilet:ti,ab OR grepid:ti,ab OR zopya:ti,ab OR zylagren:ti,ab OR zyllt:ti,ab OR cangrelor:ti,ab OR elinogrel:ti,ab OR prasugrel:ti,ab OR regrelor:ti,ab OR selatogrel:ti,ab OR ticagrelor:ti,ab OR effient:ti,ab OR efient:ti,ab OR brilinta:ti,ab OR brilique:ti,ab OR possia:ti,ab OR p2y12:ti,ab OR thienopyridine:ti,ab) OR 'purinergic p2y receptor antagonist'/exp | 107029 |
| #2 | 'purinergic p2y receptor antagonist'/exp | 70343 |
| #1 | antiplatelet:ti,ab OR clopidogrel:ti,ab OR ticlopidine:ti,ab OR plavix:ti,ab OR rivaroxaban:ti,ab OR clopilet:ti,ab OR grepid:ti,ab OR zopya:ti,ab OR zylagren:ti,ab OR zyllt:ti,ab OR cangrelor:ti,ab OR elinogrel:ti,ab OR prasugrel:ti,ab OR regrelor:ti,ab OR selatogrel:ti,ab OR ticagrelor:ti,ab OR effient:ti,ab OR efient:ti,ab OR brilinta:ti,ab OR brilique:ti,ab OR possia:ti,ab OR p2y12:ti,ab OR thienopyridine:ti,ab | 69462 |

## PubMed

| Search number | Query | Search Details | Results |
| --- | --- | --- | --- |
| 37 | #35 NOT #36 | ((((((("drug regimen"[Title/Abstract] OR "combination"[Title/Abstract]) OR "dual"[Title/Abstract]) OR "drug therapy, combination"[MeSH Terms]) AND ((((((((((((((((((((((((("Antiplatelet"[Title/Abstract] OR "Clopidogrel"[Title/Abstract]) OR "Ticlopidine"[Title/Abstract]) OR "Plavix"[Title/Abstract]) OR "Prasugrel"[Title/Abstract]) OR "rivaroxaban"[Title/Abstract]) ) ) ) ) ) OR "cangrelor"[Title/Abstract]) OR "elinogrel"[Title/Abstract]) ) OR "selatogrel"[Title/Abstract]) OR "ticagrelor"[Title/Abstract]) OR "Effient"[Title/Abstract]) OR "Efient"[Title/Abstract]) OR "brilinta"[Title/Abstract]) OR "brilique"[Title/Abstract]) OR "possia"[Title/Abstract]) OR "p2y12"[Title/Abstract]) OR "thienopyridine"[Title/Abstract]) OR ((("Thiophenes"[MeSH Terms] OR "Purinergic P2 Receptor Antagonists"[MeSH Terms]) OR "Purinergic P2 Receptor Antagonists"[Pharmacological Action]) OR "Adenosine"[MeSH Terms])) AND (((((((((((((((((((((("Acid"[Title/Abstract]) OR ("acetylsalycylic"[All Fields] AND "Acid"[Title/Abstract])) OR "aspirin"[Title/Abstract]) OR "aspirine"[Title/Abstract]) OR "acetylsalicylic acid"[Title/Abstract]) OR "Acylpyrin"[Title/Abstract]) ) OR "Colfarit"[Title/Abstract]) ) OR "Easprin"[Title/Abstract]) OR "Ecotrin"[Title/Abstract]) OR "Endosprin"[Title/Abstract]) OR "Magnecyl"[Title/Abstract]) OR "Micristin"[Title/Abstract]) OR "Polopirin"[Title/Abstract]) OR "Polopiryna"[Title/Abstract]) OR "Solprin"[Title/Abstract]) OR "Solupsan"[Title/Abstract]) OR "Zorprin"[Title/Abstract]) OR "Acetysal"[Title/Abstract]) OR "cyclooxygenase inhibitor"[Title/Abstract]) OR "aspirin"[MeSH Terms])) OR ((((((((((((((((((((((("Acid"[Title/Abstract]) OR ("acetylsalycylic"[All Fields] AND "Acid"[Title/Abstract])) OR "aspirin"[Title/Abstract]) OR "aspirine"[Title/Abstract]) OR "acetylsalicylic acid"[Title/Abstract]) OR "Acylpyrin"[Title/Abstract]) ) OR "Colfarit"[Title/Abstract]) ) OR "Easprin"[Title/Abstract]) OR "Ecotrin"[Title/Abstract]) OR "Endosprin"[Title/Abstract]) OR "Magnecyl"[Title/Abstract]) OR "Micristin"[Title/Abstract]) OR "Polopirin"[Title/Abstract]) OR "Polopiryna"[Title/Abstract]) OR "Solprin"[Title/Abstract]) OR "Solupsan"[Title/Abstract]) OR "Zorprin"[Title/Abstract]) OR "Acetysal"[Title/Abstract]) OR "cyclooxygenase inhibitor"[Title/Abstract]) OR "aspirin"[MeSH Terms]) AND ((((((((((("anticoagul*"[Title/Abstract] OR "Antithrombin"[Title/Abstract]) OR "factor xa inhibitors"[Title/Abstract]) OR "Xarelto"[Title/Abstract]) OR "rivaroxaban"[Title/Abstract]) OR "apixaban"[Title/Abstract]) OR "Pradaxa"[Title/Abstract]) OR "Eliquis"[Title/Abstract]) OR "dabigatran"[Title/Abstract]) OR "NOAC"[Title/Abstract]) OR "DOAC"[Title/Abstract]) OR "hematologic agents"[MeSH Terms])))) AND ((((((((((((((((((((((((((("cardiovascular disease"[Title/Abstract] OR "vascular disease"[Title/Abstract]) OR "heart disease"[Title/Abstract]) OR "cardiac disorder"[Title/Abstract]) OR "heart disorder"[Title/Abstract]) OR "Coronary"[Title/Abstract]) OR "angina"[Title/Abstract]) OR "peripheral disease"[Title/Abstract]) OR "myocardial reperfusion"[Title/Abstract]) OR "arterial obstructive diseases"[Title/Abstract]) OR "peripheral angiopathy"[Title/Abstract]) OR "major adverse cardiovascular event"[Title/Abstract]) OR "cardiopathy"[Title/Abstract]) OR "angiopathy"[Title/Abstract]) OR "blood vessel disease"[Title/Abstract]) OR "vasculopathy"[Title/Abstract]) OR "heart attack"[Title/Abstract]) OR "cardiovascular stroke"[Title/Abstract]) OR "atherosclero*"[Title/Abstract]) OR "isch*"[Title/Abstract]) OR "infarction"[Title/Abstract]) OR "STEMI"[Title/Abstract]) OR "thrombosis"[Title/Abstract]) OR "thrombotic"[Title/Abstract]) OR "stable"[Title/Abstract]) OR "cardiovascular diseases"[MeSH Terms]) OR ((("percutaneous coronary intervention"[Title/Abstract] OR "percutaneous coronary revascularizations"[Title/Abstract]) OR "percutaneous transluminal angioplasty"[Title/Abstract]) OR "endovascular procedures"[MeSH Terms])) OR ((((("drug"[All Fields] AND (((((((((("elutable"[All Fields] OR "elutant"[All Fields]) OR "elute"[All Fields]) OR "eluted"[All Fields]) OR "elutent"[All Fields]) OR "eluter"[All Fields]) OR "eluters"[All Fields]) OR "elutes"[All Fields]) OR "eluting"[All Fields]) OR "elution"[All Fields]) OR "elutions"[All Fields])) AND "cardiovascular stent"[Title/Abstract]) OR "drug eluting coronary artery stent"[Title/Abstract]) OR "drug eluting stent"[Title/Abstract]) OR "drug-eluting stents"[MeSH Terms]))) AND ((((("Long-Term"[Title/Abstract] OR "chronic treatment"[Title/Abstract]) OR "life support care"[Title/Abstract]) OR "Long-Term"[Title/Abstract]) OR "long-term care"[MeSH Terms]) OR (((((("duration"[Title/Abstract] OR "length"[Title/Abstract]) OR "extended"[Title/Abstract]) OR "continue"[Title/Abstract]) OR "follow"[Title/Abstract]) OR "beyond"[Title/Abstract]) OR "duration of therapy"[MeSH Terms]))) AND ((((((("randomized controlled trial"[Publication Type] OR "controlled clinical trial"[Publication Type]) OR "randomized"[Title/Abstract]) OR "placebo"[Title/Abstract]) OR "clinical trials as topic"[MeSH Terms:noexp]) OR "randomly"[Title/Abstract]) OR "trial"[Title]) NOT ("animals"[MeSH Terms] NOT ("humans"[MeSH Terms] AND "animals"[MeSH Terms])))) NOT "systematic review"[Publication Type] | 891 |
| 36 | Systematic Review [Publication Type] | "systematic review"[Publication Type] | 125,611 |
| 35 | #33 AND #34 | (((((("drug regimen"[Title/Abstract] OR "combination"[Title/Abstract]) OR "dual"[Title/Abstract]) OR "drug therapy, combination"[MeSH Terms]) AND ((((((((((((((((((((((((("Antiplatelet"[Title/Abstract] OR "Clopidogrel"[Title/Abstract]) OR "Ticlopidine"[Title/Abstract]) OR "Plavix"[Title/Abstract]) OR "Prasugrel"[Title/Abstract]) OR "rivaroxaban"[Title/Abstract]) ) ) ) ) ) OR "cangrelor"[Title/Abstract]) OR "elinogrel"[Title/Abstract]) ) OR "selatogrel"[Title/Abstract]) OR "ticagrelor"[Title/Abstract]) OR "Effient"[Title/Abstract]) OR "Efient"[Title/Abstract]) OR "brilinta"[Title/Abstract]) OR "brilique"[Title/Abstract]) OR "possia"[Title/Abstract]) OR "p2y12"[Title/Abstract]) OR "thienopyridine"[Title/Abstract]) OR ((("Thiophenes"[MeSH Terms] OR "Purinergic P2 Receptor Antagonists"[MeSH Terms]) OR "Purinergic P2 Receptor Antagonists"[Pharmacological Action]) OR "Adenosine"[MeSH Terms])) AND (((((((((((((((((((((("Acid"[Title/Abstract]) OR ("acetylsalycylic"[All Fields] AND "Acid"[Title/Abstract])) OR "aspirin"[Title/Abstract]) OR "aspirine"[Title/Abstract]) OR "acetylsalicylic acid"[Title/Abstract]) OR "Acylpyrin"[Title/Abstract]) ) OR "Colfarit"[Title/Abstract]) ) OR "Easprin"[Title/Abstract]) OR "Ecotrin"[Title/Abstract]) OR "Endosprin"[Title/Abstract]) OR "Magnecyl"[Title/Abstract]) OR "Micristin"[Title/Abstract]) OR "Polopirin"[Title/Abstract]) OR "Polopiryna"[Title/Abstract]) OR "Solprin"[Title/Abstract]) OR "Solupsan"[Title/Abstract]) OR "Zorprin"[Title/Abstract]) OR "Acetysal"[Title/Abstract]) OR "cyclooxygenase inhibitor"[Title/Abstract]) OR "aspirin"[MeSH Terms])) OR ((((((((((((((((((((((("Acid"[Title/Abstract]) OR ("acetylsalycylic"[All Fields] AND "Acid"[Title/Abstract])) OR "aspirin"[Title/Abstract]) OR "aspirine"[Title/Abstract]) OR "acetylsalicylic acid"[Title/Abstract]) OR "Acylpyrin"[Title/Abstract]) ) OR "Colfarit"[Title/Abstract]) ) OR "Easprin"[Title/Abstract]) OR "Ecotrin"[Title/Abstract]) OR "Endosprin"[Title/Abstract]) OR "Magnecyl"[Title/Abstract]) OR "Micristin"[Title/Abstract]) OR "Polopirin"[Title/Abstract]) OR "Polopiryna"[Title/Abstract]) OR "Solprin"[Title/Abstract]) OR "Solupsan"[Title/Abstract]) OR "Zorprin"[Title/Abstract]) OR "Acetysal"[Title/Abstract]) OR "cyclooxygenase inhibitor"[Title/Abstract]) OR "aspirin"[MeSH Terms]) AND ((((((((((("anticoagul*"[Title/Abstract] OR "Antithrombin"[Title/Abstract]) OR "factor xa inhibitors"[Title/Abstract]) OR "Xarelto"[Title/Abstract]) OR "rivaroxaban"[Title/Abstract]) OR "apixaban"[Title/Abstract]) OR "Pradaxa"[Title/Abstract]) OR "Eliquis"[Title/Abstract]) OR "dabigatran"[Title/Abstract]) OR "NOAC"[Title/Abstract]) OR "DOAC"[Title/Abstract]) OR "hematologic agents"[MeSH Terms])))) AND ((((((((((((((((((((((((((("cardiovascular disease"[Title/Abstract] OR "vascular disease"[Title/Abstract]) OR "heart disease"[Title/Abstract]) OR "cardiac disorder"[Title/Abstract]) OR "heart disorder"[Title/Abstract]) OR "Coronary"[Title/Abstract]) OR "angina"[Title/Abstract]) OR "peripheral disease"[Title/Abstract]) OR "myocardial reperfusion"[Title/Abstract]) OR "arterial obstructive diseases"[Title/Abstract]) OR "peripheral angiopathy"[Title/Abstract]) OR "major adverse cardiovascular event"[Title/Abstract]) OR "cardiopathy"[Title/Abstract]) OR "angiopathy"[Title/Abstract]) OR "blood vessel disease"[Title/Abstract]) OR "vasculopathy"[Title/Abstract]) OR "heart attack"[Title/Abstract]) OR "cardiovascular stroke"[Title/Abstract]) OR "atherosclero*"[Title/Abstract]) OR "isch*"[Title/Abstract]) OR "infarction"[Title/Abstract]) OR "STEMI"[Title/Abstract]) OR "thrombosis"[Title/Abstract]) OR "thrombotic"[Title/Abstract]) OR "stable"[Title/Abstract]) OR "cardiovascular diseases"[MeSH Terms]) OR ((("percutaneous coronary intervention"[Title/Abstract] OR "percutaneous coronary revascularizations"[Title/Abstract]) OR "percutaneous transluminal angioplasty"[Title/Abstract]) OR "endovascular procedures"[MeSH Terms])) OR ((((("drug"[All Fields] AND (((((((((("elutable"[All Fields] OR "elutant"[All Fields]) OR "elute"[All Fields]) OR "eluted"[All Fields]) OR "elutent"[All Fields]) OR "eluter"[All Fields]) OR "eluters"[All Fields]) OR "elutes"[All Fields]) OR "eluting"[All Fields]) OR "elution"[All Fields]) OR "elutions"[All Fields])) AND "cardiovascular stent"[Title/Abstract]) OR "drug eluting coronary artery stent"[Title/Abstract]) OR "drug eluting stent"[Title/Abstract]) OR "drug-eluting stents"[MeSH Terms]))) AND ((((("Long-Term"[Title/Abstract] OR "chronic treatment"[Title/Abstract]) OR "life support care"[Title/Abstract]) OR "Long-Term"[Title/Abstract]) OR "long-term care"[MeSH Terms]) OR (((((("duration"[Title/Abstract] OR "length"[Title/Abstract]) OR "extended"[Title/Abstract]) OR "continue"[Title/Abstract]) OR "follow"[Title/Abstract]) OR "beyond"[Title/Abstract]) OR "duration of therapy"[MeSH Terms]))) AND ((((((("randomized controlled trial"[Publication Type] OR "controlled clinical trial"[Publication Type]) OR "randomized"[Title/Abstract]) OR "placebo"[Title/Abstract]) OR "clinical trials as topic"[MeSH Terms:noexp]) OR "randomly"[Title/Abstract]) OR "trial"[Title]) NOT ("animals"[MeSH Terms] NOT ("humans"[MeSH Terms] AND "animals"[MeSH Terms]))) | 940 |
| 34 | (randomized controlled trial[pt] OR controlled clinical trial[pt] OR randomized[tiab] OR placebo[tiab] OR clinical trials as topic[mesh:noexp] OR randomly[tiab] OR trial[ti]) NOT (animals [mh] NOT (humans [mh] AND animals[mh])) | (((((("randomized controlled trial"[Publication Type] OR "controlled clinical trial"[Publication Type]) OR "randomized"[Title/Abstract]) OR "placebo"[Title/Abstract]) OR "clinical trials as topic"[MeSH Terms:noexp]) OR "randomly"[Title/Abstract]) OR "trial"[Title]) NOT ("animals"[MeSH Terms] NOT ("humans"[MeSH Terms] AND "animals"[MeSH Terms])) | 1,185,626 |
| 33 | #12 AND #30 AND #31 AND #32 | ((((("drug regimen"[Title/Abstract] OR "combination"[Title/Abstract]) OR "dual"[Title/Abstract]) OR "drug therapy, combination"[MeSH Terms]) AND ((((((((((((((((((((((((("Antiplatelet"[Title/Abstract] OR "Clopidogrel"[Title/Abstract]) OR "Ticlopidine"[Title/Abstract]) OR "Plavix"[Title/Abstract]) OR "Prasugrel"[Title/Abstract]) OR "rivaroxaban"[Title/Abstract]) ) ) ) ) ) OR "cangrelor"[Title/Abstract]) OR "elinogrel"[Title/Abstract]) ) OR "selatogrel"[Title/Abstract]) OR "ticagrelor"[Title/Abstract]) OR "Effient"[Title/Abstract]) OR "Efient"[Title/Abstract]) OR "brilinta"[Title/Abstract]) OR "brilique"[Title/Abstract]) OR "possia"[Title/Abstract]) OR "p2y12"[Title/Abstract]) OR "thienopyridine"[Title/Abstract]) OR ((("Thiophenes"[MeSH Terms] OR "Purinergic P2 Receptor Antagonists"[MeSH Terms]) OR "Purinergic P2 Receptor Antagonists"[Pharmacological Action]) OR "Adenosine"[MeSH Terms])) AND (((((((((((((((((((((("Acid"[Title/Abstract]) OR ("acetylsalycylic"[All Fields] AND "Acid"[Title/Abstract])) OR "aspirin"[Title/Abstract]) OR "aspirine"[Title/Abstract]) OR "acetylsalicylic acid"[Title/Abstract]) OR "Acylpyrin"[Title/Abstract]) ) OR "Colfarit"[Title/Abstract]) ) OR "Easprin"[Title/Abstract]) OR "Ecotrin"[Title/Abstract]) OR "Endosprin"[Title/Abstract]) OR "Magnecyl"[Title/Abstract]) OR "Micristin"[Title/Abstract]) OR "Polopirin"[Title/Abstract]) OR "Polopiryna"[Title/Abstract]) OR "Solprin"[Title/Abstract]) OR "Solupsan"[Title/Abstract]) OR "Zorprin"[Title/Abstract]) OR "Acetysal"[Title/Abstract]) OR "cyclooxygenase inhibitor"[Title/Abstract]) OR "aspirin"[MeSH Terms])) OR ((((((((((((((((((((((("Acid"[Title/Abstract]) OR ("acetylsalycylic"[All Fields] AND "Acid"[Title/Abstract])) OR "aspirin"[Title/Abstract]) OR "aspirine"[Title/Abstract]) OR "acetylsalicylic acid"[Title/Abstract]) OR "Acylpyrin"[Title/Abstract]) ) OR "Colfarit"[Title/Abstract]) ) OR "Easprin"[Title/Abstract]) OR "Ecotrin"[Title/Abstract]) OR "Endosprin"[Title/Abstract]) OR "Magnecyl"[Title/Abstract]) OR "Micristin"[Title/Abstract]) OR "Polopirin"[Title/Abstract]) OR "Polopiryna"[Title/Abstract]) OR "Solprin"[Title/Abstract]) OR "Solupsan"[Title/Abstract]) OR "Zorprin"[Title/Abstract]) OR "Acetysal"[Title/Abstract]) OR "cyclooxygenase inhibitor"[Title/Abstract]) OR "aspirin"[MeSH Terms]) AND ((((((((((("anticoagul*"[Title/Abstract] OR "Antithrombin"[Title/Abstract]) OR "factor xa inhibitors"[Title/Abstract]) OR "Xarelto"[Title/Abstract]) OR "rivaroxaban"[Title/Abstract]) OR "apixaban"[Title/Abstract]) OR "Pradaxa"[Title/Abstract]) OR "Eliquis"[Title/Abstract]) OR "dabigatran"[Title/Abstract]) OR "NOAC"[Title/Abstract]) OR "DOAC"[Title/Abstract]) OR "hematologic agents"[MeSH Terms])))) AND ((((((((((((((((((((((((((("cardiovascular disease"[Title/Abstract] OR "vascular disease"[Title/Abstract]) OR "heart disease"[Title/Abstract]) OR "cardiac disorder"[Title/Abstract]) OR "heart disorder"[Title/Abstract]) OR "Coronary"[Title/Abstract]) OR "angina"[Title/Abstract]) OR "peripheral disease"[Title/Abstract]) OR "myocardial reperfusion"[Title/Abstract]) OR "arterial obstructive diseases"[Title/Abstract]) OR "peripheral angiopathy"[Title/Abstract]) OR "major adverse cardiovascular event"[Title/Abstract]) OR "cardiopathy"[Title/Abstract]) OR "angiopathy"[Title/Abstract]) OR "blood vessel disease"[Title/Abstract]) OR "vasculopathy"[Title/Abstract]) OR "heart attack"[Title/Abstract]) OR "cardiovascular stroke"[Title/Abstract]) OR "atherosclero*"[Title/Abstract]) OR "isch*"[Title/Abstract]) OR "infarction"[Title/Abstract]) OR "STEMI"[Title/Abstract]) OR "thrombosis"[Title/Abstract]) OR "thrombotic"[Title/Abstract]) OR "stable"[Title/Abstract]) OR "cardiovascular diseases"[MeSH Terms]) OR ((("percutaneous coronary intervention"[Title/Abstract] OR "percutaneous coronary revascularizations"[Title/Abstract]) OR "percutaneous transluminal angioplasty"[Title/Abstract]) OR "endovascular procedures"[MeSH Terms])) OR ((((("drug"[All Fields] AND (((((((((("elutable"[All Fields] OR "elutant"[All Fields]) OR "elute"[All Fields]) OR "eluted"[All Fields]) OR "elutent"[All Fields]) OR "eluter"[All Fields]) OR "eluters"[All Fields]) OR "elutes"[All Fields]) OR "eluting"[All Fields]) OR "elution"[All Fields]) OR "elutions"[All Fields])) AND "cardiovascular stent"[Title/Abstract]) OR "drug eluting coronary artery stent"[Title/Abstract]) OR "drug eluting stent"[Title/Abstract]) OR "drug-eluting stents"[MeSH Terms]))) AND ((((("Long-Term"[Title/Abstract] OR "chronic treatment"[Title/Abstract]) OR "life support care"[Title/Abstract]) OR "Long-Term"[Title/Abstract]) OR "long-term care"[MeSH Terms]) OR (((((("duration"[Title/Abstract] OR "length"[Title/Abstract]) OR "extended"[Title/Abstract]) OR "continue"[Title/Abstract]) OR "follow"[Title/Abstract]) OR "beyond"[Title/Abstract]) OR "duration of therapy"[MeSH Terms])) | 2,331 |
| 32 | #24 OR #27 | "Long-Term"[Title/Abstract] OR "chronic treatment"[Title/Abstract] OR "life support care"[Title/Abstract] OR "Long-Term"[Title/Abstract] OR "long-term care"[MeSH Terms] OR "duration"[Title/Abstract] OR "length"[Title/Abstract] OR "extended"[Title/Abstract] OR "continue"[Title/Abstract] OR "follow"[Title/Abstract] OR "beyond"[Title/Abstract] OR "duration of therapy"[MeSH Terms] | 3,150,424 |
| 31 | #15 OR #18 OR #21 | (((((((((((((((((((((((((("cardiovascular disease"[Title/Abstract] OR "vascular disease"[Title/Abstract]) OR "heart disease"[Title/Abstract]) OR "cardiac disorder"[Title/Abstract]) OR "heart disorder"[Title/Abstract]) OR "Coronary"[Title/Abstract]) OR "angina"[Title/Abstract]) OR "peripheral disease"[Title/Abstract]) OR "myocardial reperfusion"[Title/Abstract]) OR "arterial obstructive diseases"[Title/Abstract]) OR "peripheral angiopathy"[Title/Abstract]) OR "major adverse cardiovascular event"[Title/Abstract]) OR "cardiopathy"[Title/Abstract]) OR "angiopathy"[Title/Abstract]) OR "blood vessel disease"[Title/Abstract]) OR "vasculopathy"[Title/Abstract]) OR "heart attack"[Title/Abstract]) OR "cardiovascular stroke"[Title/Abstract]) OR "atherosclero*"[Title/Abstract]) OR "isch*"[Title/Abstract]) OR "infarction"[Title/Abstract]) OR "STEMI"[Title/Abstract]) OR "thrombosis"[Title/Abstract]) OR "thrombotic"[Title/Abstract]) OR "stable"[Title/Abstract]) OR "cardiovascular diseases"[MeSH Terms]) OR ((("percutaneous coronary intervention"[Title/Abstract] OR "percutaneous coronary revascularizations"[Title/Abstract]) OR "percutaneous transluminal angioplasty"[Title/Abstract]) OR "endovascular procedures"[MeSH Terms])) OR ((((("drug"[All Fields] AND (((((((((("elutable"[All Fields] OR "elutant"[All Fields]) OR "elute"[All Fields]) OR "eluted"[All Fields]) OR "elutent"[All Fields]) OR "eluter"[All Fields]) OR "eluters"[All Fields]) OR "elutes"[All Fields]) OR "eluting"[All Fields]) OR "elution"[All Fields]) OR "elutions"[All Fields])) AND "cardiovascular stent"[Title/Abstract]) OR "drug eluting coronary artery stent"[Title/Abstract]) OR "drug eluting stent"[Title/Abstract]) OR "drug-eluting stents"[MeSH Terms]) | 3,256,687 |
| 30 | #28 OR #29 | (((((((((((((((((((((((("Antiplatelet"[Title/Abstract] OR "Clopidogrel"[Title/Abstract]) OR "Ticlopidine"[Title/Abstract]) OR "Plavix"[Title/Abstract]) OR "Prasugrel"[Title/Abstract]) OR "rivaroxaban"[Title/Abstract]) ) ) ) ) ) OR "cangrelor"[Title/Abstract]) OR "elinogrel"[Title/Abstract]) ) OR "selatogrel"[Title/Abstract]) OR "ticagrelor"[Title/Abstract]) OR "Effient"[Title/Abstract]) OR "Efient"[Title/Abstract]) OR "brilinta"[Title/Abstract]) OR "brilique"[Title/Abstract]) OR "possia"[Title/Abstract]) OR "p2y12"[Title/Abstract]) OR "thienopyridine"[Title/Abstract]) OR ((("Thiophenes"[MeSH Terms] OR "Purinergic P2 Receptor Antagonists"[MeSH Terms]) OR "Purinergic P2 Receptor Antagonists"[Pharmacological Action]) OR "Adenosine"[MeSH Terms])) AND (((((((((((((((((((((("Acid"[Title/Abstract]) OR ("acetylsalycylic"[All Fields] AND "Acid"[Title/Abstract])) OR "aspirin"[Title/Abstract]) OR "aspirine"[Title/Abstract]) OR "acetylsalicylic acid"[Title/Abstract]) OR "Acylpyrin"[Title/Abstract]) ) OR "Colfarit"[Title/Abstract]) ) OR "Easprin"[Title/Abstract]) OR "Ecotrin"[Title/Abstract]) OR "Endosprin"[Title/Abstract]) OR "Magnecyl"[Title/Abstract]) OR "Micristin"[Title/Abstract]) OR "Polopirin"[Title/Abstract]) OR "Polopiryna"[Title/Abstract]) OR "Solprin"[Title/Abstract]) OR "Solupsan"[Title/Abstract]) OR "Zorprin"[Title/Abstract]) OR "Acetysal"[Title/Abstract]) OR "cyclooxygenase inhibitor"[Title/Abstract]) OR "aspirin"[MeSH Terms])) OR ((((((((((((((((((((((("Acid"[Title/Abstract]) OR ("acetylsalycylic"[All Fields] AND "Acid"[Title/Abstract])) OR "aspirin"[Title/Abstract]) OR "aspirine"[Title/Abstract]) OR "acetylsalicylic acid"[Title/Abstract]) OR "Acylpyrin"[Title/Abstract]) ) OR "Colfarit"[Title/Abstract]) ) OR "Easprin"[Title/Abstract]) OR "Ecotrin"[Title/Abstract]) OR "Endosprin"[Title/Abstract]) OR "Magnecyl"[Title/Abstract]) OR "Micristin"[Title/Abstract]) OR "Polopirin"[Title/Abstract]) OR "Polopiryna"[Title/Abstract]) OR "Solprin"[Title/Abstract]) OR "Solupsan"[Title/Abstract]) OR "Zorprin"[Title/Abstract]) OR "Acetysal"[Title/Abstract]) OR "cyclooxygenase inhibitor"[Title/Abstract]) OR "aspirin"[MeSH Terms]) AND ((((((((((("anticoagul*"[Title/Abstract] OR "Antithrombin"[Title/Abstract]) OR "factor xa inhibitors"[Title/Abstract]) OR "Xarelto"[Title/Abstract]) OR "rivaroxaban"[Title/Abstract]) OR "apixaban"[Title/Abstract]) OR "Pradaxa"[Title/Abstract]) OR "Eliquis"[Title/Abstract]) OR "dabigatran"[Title/Abstract]) OR "NOAC"[Title/Abstract]) OR "DOAC"[Title/Abstract]) OR "hematologic agents"[MeSH Terms])) | 48,142 |
| 29 | #6 AND #9 | (((((((((((((((((((((("Acid"[Title/Abstract]) OR ("acetylsalycylic"[All Fields] AND "Acid"[Title/Abstract])) OR "aspirin"[Title/Abstract]) OR "aspirine"[Title/Abstract]) OR "acetylsalicylic acid"[Title/Abstract]) OR "Acylpyrin"[Title/Abstract]) ) OR "Colfarit"[Title/Abstract]) ) OR "Easprin"[Title/Abstract]) OR "Ecotrin"[Title/Abstract]) OR "Endosprin"[Title/Abstract]) OR "Magnecyl"[Title/Abstract]) OR "Micristin"[Title/Abstract]) OR "Polopirin"[Title/Abstract]) OR "Polopiryna"[Title/Abstract]) OR "Solprin"[Title/Abstract]) OR "Solupsan"[Title/Abstract]) OR "Zorprin"[Title/Abstract]) OR "Acetysal"[Title/Abstract]) OR "cyclooxygenase inhibitor"[Title/Abstract]) OR "aspirin"[MeSH Terms]) AND ((((((((((("anticoagul*"[Title/Abstract] OR "Antithrombin"[Title/Abstract]) OR "factor xa inhibitors"[Title/Abstract]) OR "Xarelto"[Title/Abstract]) OR "rivaroxaban"[Title/Abstract]) OR "apixaban"[Title/Abstract]) OR "Pradaxa"[Title/Abstract]) OR "Eliquis"[Title/Abstract]) OR "dabigatran"[Title/Abstract]) OR "NOAC"[Title/Abstract]) OR "DOAC"[Title/Abstract]) OR "hematologic agents"[MeSH Terms]) | 33,143 |
| 28 | #3 AND #6 | ((((((((((((((((((((((("Antiplatelet"[Title/Abstract] OR "Clopidogrel"[Title/Abstract]) OR "Ticlopidine"[Title/Abstract]) OR "Plavix"[Title/Abstract]) OR "Prasugrel"[Title/Abstract]) OR "rivaroxaban"[Title/Abstract]) ) ) ) ) ) OR "cangrelor"[Title/Abstract]) OR "elinogrel"[Title/Abstract]) ) OR "selatogrel"[Title/Abstract]) OR "ticagrelor"[Title/Abstract]) OR "Effient"[Title/Abstract]) OR "Efient"[Title/Abstract]) OR "brilinta"[Title/Abstract]) OR "brilique"[Title/Abstract]) OR "possia"[Title/Abstract]) OR "p2y12"[Title/Abstract]) OR "thienopyridine"[Title/Abstract]) OR ((("Thiophenes"[MeSH Terms] OR "Purinergic P2 Receptor Antagonists"[MeSH Terms]) OR "Purinergic P2 Receptor Antagonists"[Pharmacological Action]) OR "Adenosine"[MeSH Terms])) AND (((((((((((((((((((((("Acid"[Title/Abstract]) OR ("acetylsalycylic"[All Fields] AND "Acid"[Title/Abstract])) OR "aspirin"[Title/Abstract]) OR "aspirine"[Title/Abstract]) OR "acetylsalicylic acid"[Title/Abstract]) OR "Acylpyrin"[Title/Abstract]) ) OR "Colfarit"[Title/Abstract]) ) OR "Easprin"[Title/Abstract]) OR "Ecotrin"[Title/Abstract]) OR "Endosprin"[Title/Abstract]) OR "Magnecyl"[Title/Abstract]) OR "Micristin"[Title/Abstract]) OR "Polopirin"[Title/Abstract]) OR "Polopiryna"[Title/Abstract]) OR "Solprin"[Title/Abstract]) OR "Solupsan"[Title/Abstract]) OR "Zorprin"[Title/Abstract]) OR "Acetysal"[Title/Abstract]) OR "cyclooxygenase inhibitor"[Title/Abstract]) OR "aspirin"[MeSH Terms]) | 26,788 |
| 27 | #25 OR #26 | "duration"[Title/Abstract] OR "length"[Title/Abstract] OR "extended"[Title/Abstract] OR "continue"[Title/Abstract] OR "follow"[Title/Abstract] OR "beyond"[Title/Abstract] OR "duration of therapy"[MeSH Terms] | 2,587,680 |
| 26 | duration of therapy[Mesh] | "duration of therapy"[MeSH Terms] | 51 |
| 25 | duration[Title/Abstract] OR length[Title/Abstract] OR extended[Title/Abstract] OR continue[Title/Abstract] OR follow[Title/Abstract] OR beyond[Title/Abstract] | "duration"[Title/Abstract] OR "length"[Title/Abstract] OR "extended"[Title/Abstract] OR "continue"[Title/Abstract] OR "follow"[Title/Abstract] OR "beyond"[Title/Abstract] | 2,587,668 |
| 24 | #22 OR #23 | "Long-Term"[Title/Abstract] OR "chronic treatment"[Title/Abstract] OR "life support care"[Title/Abstract] OR "Long-Term"[Title/Abstract] OR "long-term care"[MeSH Terms] | 803,525 |
| 23 | Long Term Care[Mesh] | "long-term care"[MeSH Terms] | 25,685 |
| 22 | Long-Term[Title/Abstract] OR chronic treatment[Title/Abstract] OR life support care[Title/Abstract] OR long term[Title/Abstract] | "Long-Term"[Title/Abstract] OR "chronic treatment"[Title/Abstract] OR "life support care"[Title/Abstract] OR "Long-Term"[Title/Abstract] | 793,718 |
| 21 | #19 OR #20 | (((("drug"[All Fields] AND (((((((((("elutable"[All Fields] OR "elutant"[All Fields]) OR "elute"[All Fields]) OR "eluted"[All Fields]) OR "elutent"[All Fields]) OR "eluter"[All Fields]) OR "eluters"[All Fields]) OR "elutes"[All Fields]) OR "eluting"[All Fields]) OR "elution"[All Fields]) OR "elutions"[All Fields])) AND "cardiovascular stent"[Title/Abstract]) OR "drug eluting coronary artery stent"[Title/Abstract]) OR "drug eluting stent"[Title/Abstract]) OR "drug-eluting stents"[MeSH Terms] | 13,104 |
| 20 | drug eluting stent[Mesh] | "drug-eluting stents"[MeSH Terms] | 11,186 |
| 19 | drug eluting cardiovascular stent[Title/Abstract] OR drug eluting coronary artery stent[Title/Abstract] OR drug eluting stent[Title/Abstract] | ((("drug"[All Fields] AND (((((((((("elutable"[All Fields] OR "elutant"[All Fields]) OR "elute"[All Fields]) OR "eluted"[All Fields]) OR "elutent"[All Fields]) OR "eluter"[All Fields]) OR "eluters"[All Fields]) OR "elutes"[All Fields]) OR "eluting"[All Fields]) OR "elution"[All Fields]) OR "elutions"[All Fields])) AND "cardiovascular stent"[Title/Abstract]) OR "drug eluting coronary artery stent"[Title/Abstract]) OR "drug eluting stent"[Title/Abstract] | 5,360 |
| 18 | #16 OR #17 | "percutaneous coronary intervention"[Title/Abstract] OR "percutaneous coronary revascularizations"[Title/Abstract] OR "percutaneous transluminal angioplasty"[Title/Abstract] OR "endovascular procedures"[MeSH Terms] | 131,902 |
| 17 | Endovascular Procedures[Mesh] | "endovascular procedures"[MeSH Terms] | 119,752 |
| 16 | percutaneous coronary intervention[Title/Abstract] OR Percutaneous Coronary Revascularizations[Title/Abstract] OR percutaneous transluminal angioplasty[Title/Abstract] | "percutaneous coronary intervention"[Title/Abstract] OR "percutaneous coronary revascularizations"[Title/Abstract] OR "percutaneous transluminal angioplasty"[Title/Abstract] | 36,042 |
| 15 | #13 OR #14 | "cardiovascular disease"[Title/Abstract] OR "vascular disease"[Title/Abstract] OR "heart disease"[Title/Abstract] OR "cardiac disorder"[Title/Abstract] OR "heart disorder"[Title/Abstract] OR "coronary"[Title/Abstract] OR "angina"[Title/Abstract] OR "peripheral disease"[Title/Abstract] OR "myocardial reperfusion"[Title/Abstract] OR "arterial obstructive diseases"[Title/Abstract] OR "peripheral angiopathy"[Title/Abstract] OR "major adverse cardiovascular event"[Title/Abstract] OR "cardiopathy"[Title/Abstract] OR "angiopathy"[Title/Abstract] OR "blood vessel disease"[Title/Abstract] OR "vasculopathy"[Title/Abstract] OR "heart attack"[Title/Abstract] OR "cardiovascular stroke"[Title/Abstract] OR "atherosclero*"[Title/Abstract] OR "isch*"[Title/Abstract] OR "infarction"[Title/Abstract] OR "STEMI"[Title/Abstract] OR "thrombosis"[Title/Abstract] OR "thrombotic"[Title/Abstract] OR "stable"[Title/Abstract] OR "cardiovascular diseases"[MeSH Terms] | 3,235,246 |
| 14 | cardiovascular disease[Mesh] | "cardiovascular diseases"[MeSH Terms] | 2,357,075 |
| 13 | cardiovascular disease[Title/Abstract] OR vascular disease[Title/Abstract] OR heart disease[Title/Abstract] OR cardiac disorder[Title/Abstract] OR heart disorder[Title/Abstract] OR coronary[Title/Abstract] OR angina[Title/Abstract] OR peripheral disease[Title/Abstract] OR myocardial reperfusion[Title/Abstract] OR arterial obstructive diseases[Title/Abstract] OR peripheral angiopathy[Title/Abstract] OR major adverse cardiovascular event[Title/Abstract] OR cardiopathy[Title/Abstract] OR angiopathy[Title/Abstract] OR blood vessel disease[Title/Abstract] OR vasculopathy[Title/Abstract] OR heart attack[Title/Abstract] OR cardiovascular stroke[Title/Abstract] OR atherosclero*[Title/Abstract] OR isch*[Title/Abstract] OR infarction[Title/Abstract] OR STEMI[Title/Abstract] OR thrombosis[Title/Abstract] OR thrombotic[Title/Abstract] OR stable[Title/Abstract] | "cardiovascular disease"[Title/Abstract] OR "vascular disease"[Title/Abstract] OR "heart disease"[Title/Abstract] OR "cardiac disorder"[Title/Abstract] OR "heart disorder"[Title/Abstract] OR "coronary"[Title/Abstract] OR "angina"[Title/Abstract] OR "peripheral disease"[Title/Abstract] OR "myocardial reperfusion"[Title/Abstract] OR "arterial obstructive diseases"[Title/Abstract] OR "peripheral angiopathy"[Title/Abstract] OR "major adverse cardiovascular event"[Title/Abstract] OR "cardiopathy"[Title/Abstract] OR "angiopathy"[Title/Abstract] OR "blood vessel disease"[Title/Abstract] OR "vasculopathy"[Title/Abstract] OR "heart attack"[Title/Abstract] OR "cardiovascular stroke"[Title/Abstract] OR "atherosclero*"[Title/Abstract] OR "isch*"[Title/Abstract] OR "infarction"[Title/Abstract] OR "STEMI"[Title/Abstract] OR "thrombosis"[Title/Abstract] OR "thrombotic"[Title/Abstract] OR "stable"[Title/Abstract] | 1,732,126 |
| 12 | #10 OR #11 | "drug regimen"[Title/Abstract] OR "combination"[Title/Abstract] OR "dual"[Title/Abstract] OR "drug therapy, combination"[MeSH Terms] | 1,224,241 |
| 11 | Drug therapy, combination[Mesh] | "drug therapy, combination"[MeSH Terms] | 321,022 |
| 10 | drug regimen[Title/Abstract] OR combination[Title/Abstract] OR dual[Title/Abstract] | "drug regimen"[Title/Abstract] OR "combination"[Title/Abstract] OR "dual"[Title/Abstract] | 1,001,048 |
| 9 | #7 OR #8 | "anticoagul*"[Title/Abstract] OR "Antithrombin"[Title/Abstract] OR "factor xa inhibitors"[Title/Abstract] OR "Xarelto"[Title/Abstract] OR "rivaroxaban"[Title/Abstract] OR "apixaban"[Title/Abstract] OR "Pradaxa"[Title/Abstract] OR "Eliquis"[Title/Abstract] OR "dabigatran"[Title/Abstract] OR "NOAC"[Title/Abstract] OR "DOAC"[Title/Abstract] OR "hematologic agents"[MeSH Terms] | 232,841 |
| 8 | hematologic agents[Mesh] | "hematologic agents"[MeSH Terms] | 174,555 |
| 7 | anticoagul*[Title/Abstract] OR Antithrombin[Title/Abstract] OR Factor Xa Inhibitors[Title/Abstract] OR Xarelto[Title/Abstract] OR rivaroxaban[Title/Abstract] OR apixaban[Title/Abstract] OR Pradaxa[Title/Abstract] OR Eliquis[Title/Abstract] OR dabigatran[Title/Abstract] OR NOAC[Title/Abstract] OR DOAC[Title/Abstract] | "anticoagul*"[Title/Abstract] OR "Antithrombin"[Title/Abstract] OR "factor xa inhibitors"[Title/Abstract] OR "Xarelto"[Title/Abstract] OR "rivaroxaban"[Title/Abstract] OR "apixaban"[Title/Abstract] OR "Pradaxa"[Title/Abstract] OR "Eliquis"[Title/Abstract] OR "dabigatran"[Title/Abstract] OR "NOAC"[Title/Abstract] OR "DOAC"[Title/Abstract] | 110,779 |
| 6 | #4 OR #5 | ((((((((((((((((((((("Acid"[Title/Abstract]) OR ("acetylsalycylic"[All Fields] AND "Acid"[Title/Abstract])) OR "aspirin"[Title/Abstract]) OR "aspirine"[Title/Abstract]) OR "acetylsalicylic acid"[Title/Abstract]) OR "Acylpyrin"[Title/Abstract]) ) OR "Colfarit"[Title/Abstract]) ) OR "Easprin"[Title/Abstract]) OR "Ecotrin"[Title/Abstract]) OR "Endosprin"[Title/Abstract]) OR "Magnecyl"[Title/Abstract]) OR "Micristin"[Title/Abstract]) OR "Polopirin"[Title/Abstract]) OR "Polopiryna"[Title/Abstract]) OR "Solprin"[Title/Abstract]) OR "Solupsan"[Title/Abstract]) OR "Zorprin"[Title/Abstract]) OR "Acetysal"[Title/Abstract]) OR "cyclooxygenase inhibitor"[Title/Abstract]) OR "aspirin"[MeSH Terms] | 1,647,964 |
| 5 | Aspirin[Mesh] | "aspirin"[MeSH Terms] | 44,575 |
| 4 | acetylsalycic acid[Title/Abstract] OR acetylsalycylic acid[Title/Abstract] OR aspirin[Title/Abstract] OR aspirine[Title/Abstract] OR Acetylsalicylic Acid[Title/Abstract] OR Acylpyrin[Title/Abstract] OR Aloxiprimum[Title/Abstract] OR Colfarit[Title/Abstract] OR Dispril[Title/Abstract] OR Easprin[Title/Abstract] OR Ecotrin[Title/Abstract] OR Endosprin[Title/Abstract] OR Magnecyl[Title/Abstract] OR Micristin[Title/Abstract] OR Polopirin[Title/Abstract] OR Polopiryna[Title/Abstract] OR Solprin[Title/Abstract] OR Solupsan[Title/Abstract] OR Zorprin[Title/Abstract] OR Acetysal[Title/Abstract] OR Cyclooxygenase Inhibitor[Title/Abstract] | (((((((((((((((((((("Acid"[Title/Abstract]) OR ("acetylsalycylic"[All Fields] AND "Acid"[Title/Abstract])) OR "aspirin"[Title/Abstract]) OR "aspirine"[Title/Abstract]) OR "acetylsalicylic acid"[Title/Abstract]) OR "Acylpyrin"[Title/Abstract]) ) OR "Colfarit"[Title/Abstract]) ) OR "Easprin"[Title/Abstract]) OR "Ecotrin"[Title/Abstract]) OR "Endosprin"[Title/Abstract]) OR "Magnecyl"[Title/Abstract]) OR "Micristin"[Title/Abstract]) OR "Polopirin"[Title/Abstract]) OR "Polopiryna"[Title/Abstract]) OR "Solprin"[Title/Abstract]) OR "Solupsan"[Title/Abstract]) OR "Zorprin"[Title/Abstract]) OR "Acetysal"[Title/Abstract]) OR "cyclooxygenase inhibitor"[Title/Abstract] | 1,635,338 |
| 3 | #1 OR #2 | "Antiplatelet"[Title/Abstract] OR "Clopidogrel"[Title/Abstract] OR "Ticlopidine"[Title/Abstract] OR "Plavix"[Title/Abstract] OR "Prasugrel"[Title/Abstract] OR "rivaroxaban "[Title/Abstract] OR "cangrelor"[Title/Abstract] OR "elinogrel "[Title/Abstract] OR "selatogrel"[Title/Abstract] OR "ticagrelor"[Title/Abstract] OR "Effient"[Title/Abstract] OR "Efient"[Title/Abstract] OR "brilinta"[Title/Abstract] OR "brilique"[Title/Abstract] OR "possia"[Title/Abstract] OR "p2y12"[Title/Abstract] OR "thienopyridine"[Title/Abstract] OR "Thiophenes"[MeSH Terms] OR "Purinergic P2 Receptor Antagonists"[MeSH Terms] OR "Purinergic P2 Receptor Antagonists"[Pharmacological Action] OR "Adenosine"[MeSH Terms] | 118,724 |
| 2 | ((("Thiophenes"[Mesh]) OR "Purinergic P2 Receptor Antagonists"[Mesh]) OR "Purinergic P2 Receptor Antagonists" [Pharmacological Action]) OR "Adenosine"[Mesh] | "Thiophenes"[MeSH Terms] OR "Purinergic P2 Receptor Antagonists"[MeSH Terms] OR "Purinergic P2 Receptor Antagonists"[Pharmacological Action] OR "Adenosine"[MeSH Terms] | 88,212 |
| 1 | Antiplatelet[Title/Abstract] OR Clopidogrel[Title/Abstract] OR Ticlopidine[Title/Abstract] OR Plavix[Title/Abstract] OR Prasugrel[Title/Abstract] OR rivaroxaban[Title/Abstract] OR Clopilet[Title/Abstract] OR grepid[Title/Abstract] OR zopya[Title/Abstract] OR zylagren[Title/Abstract] OR zyllt[Title/Abstract] OR cangrelor[Title/Abstract] OR elinogrel[Title/Abstract] OR regrelor[Title/Abstract] OR selatogrel[Title/Abstract] OR ticagrelor[Title/Abstract] OR Effient[Title/Abstract] OR Efient[Title/Abstract] OR brilinta[Title/Abstract] OR brilique[Title/Abstract] OR possia[Title/Abstract] OR p2y12[Title/Abstract] OR thienopyridine[Title/Abstract] | "Antiplatelet"[Title/Abstract] OR "Clopidogrel"[Title/Abstract] OR "Ticlopidine"[Title/Abstract] OR "Plavix"[Title/Abstract] OR "Prasugrel"[Title/Abstract] OR "rivaroxaban "[Title/Abstract] OR "cangrelor"[Title/Abstract] OR "elinogrel "[Title/Abstract] OR "selatogrel"[Title/Abstract] OR "ticagrelor"[Title/Abstract] OR "Effient"[Title/Abstract] OR "Efient"[Title/Abstract] OR "brilinta"[Title/Abstract] OR "brilique"[Title/Abstract] OR "possia"[Title/Abstract] OR "p2y12"[Title/Abstract] OR "thienopyridine"[Title/Abstract] | 44,092 |

## Cochrane

| ID | Search | Hits |
| --- | --- | --- |
| #1 | MeSH descriptor: [Thiophenes] explode all trees | 5193 |
| #2 | MeSH descriptor: [Purinergic P2 Receptor Antagonists] explode all trees | 366 |
| #3 | MeSH descriptor: [Adenosine] explode all trees | 1841 |
| #4 | #1 OR #2 OR #3 | 6639 |
| #5 | (Antiplatelet OR Clopidogrel OR Ticlopidine OR Plavix OR Prasugrel OR rivaroxaban OR Clopilet OR grepid OR zopya OR zylagren OR zyllt OR cangrelor OR elinogrel OR regrelor OR selatogrel OR ticagrelor OR Effient OR Efient OR brilinta OR brilique OR possia OR p2y12 OR thienopyridine):ab | 9265 |
| #6 | #4 OR #5 | 13103 |
| #7 | (acetylsalycic acid OR acetylsalycylic acid OR aspirin OR aspirine OR Acetylsalicylic Acid OR Acylpyrin OR Aloxiprimum OR Colfarit OR Dispril OR Easprin OR Ecotrin OR Endosprin OR Magnecyl OR Micristin OR Polopirin OR Polopiryna OR Solprin OR Solupsan OR Zorprin OR Acetysal OR Cyclooxygenase Inhibitor):ab | 11910 |
| #8 | MeSH descriptor: [Aspirin] explode all trees | 5781 |
| #9 | #7 OR #8 | 13176 |
| #10 | (anticoagul* OR Antithrombin OR Factor Xa Inhibitors OR Xarelto OR rivaroxaban OR apixaban OR Pradaxa OR Eliquis OR dabigatran OR NOAC OR DOAC):ab | 10983 |
| #11 | MeSH descriptor: [Hematologic Agents] explode all trees | 12386 |
| #12 | #10 OR #11 | 20509 |
| #13 | (drug regimen OR combination OR dual):ab | 110236 |
| #14 | MeSH descriptor: [Drug Therapy, Combination] explode all trees | 43658 |
| #15 | #13 OR #14 | 137939 |
| #16 | (cardiovascular disease OR vascular disease OR heart disease OR cardiac disorder OR heart disorder OR coronary OR angina OR peripheral disease OR myocardial reperfusion OR arterial obstructive diseases OR peripheral angiopathy OR major adverse cardiovascular event OR cardiopathy OR angiopathy OR blood vessel disease OR vasculopathy OR heart attack OR cardiovascular stroke OR atherosclero* OR isch* OR infarction OR STEMI OR thrombosis OR thrombotic OR stable):ti | 69866 |
| #17 | MeSH descriptor: [Cardiovascular Diseases] explode all trees | 104780 |
| #18 | (percutaneous coronary intervention OR Percutaneous Coronary Revascularizations OR percutaneous transluminal angioplasty):ab | 8780 |
| #19 | MeSH descriptor: [Endovascular Procedures] explode all trees | 8216 |
| #20 | #18 OR #19 | 13599 |
| #21 | (drug eluting cardiovascular stent OR drug eluting coronary artery stent OR drug eluting stent):ab | 2225 |
| #22 | MeSH descriptor: [Drug-Eluting Stents] explode all trees | 1340 |
| #23 | #21 OR #22 | 2881 |
| #24 | (chronic treatment OR life support care OR long term):ab | 137518 |
| #25 | MeSH descriptor: [Long-Term Care] explode all trees | 1107 |
| #26 | #24 OR #25 | 137982 |
| #27 | (duration OR length OR extended OR continue OR follow OR beyond):ab | 338960 |
| #28 | MeSH descriptor: [Duration of Therapy] explode all trees | 5 |
| #29 | #27 OR #28 | 338960 |
| #30 | #6 AND #9 | 4041 |
| #31 | #9 AND #12 | 3545 |
| #32 | #30 OR #31 | 5636 |
| #33 | #16 OR #17 | 145835 |
| #34 | #33 OR #20 OR #23 | 148768 |
| #35 | #26 OR #29 | 420761 |
| #36 | #15 AND #32 AND #34 AND #35 | 800 |

## Clinical trials

**228** Studies found for: **long term OR extended OR dual OR duration | Active, not recruiting, Completed, Terminated Studies | Studies With Results | Interventional Studies | Cardiovascular Diseases OR ischemia OR heart attack OR peripheral disease OR angina OR myocardial infraction OR stroke OR coronary artery disease OR drug eluting stent OR percutaneous coronary intervention | Aspirin OR p2y12 OR anti platelet OR anticoagulant OR prasugrel OR ticagrelor OR clopidogrel OR ticlopidine OR rivaroxaban OR apixaban OR dabigatran | Adult, Older Adult**

Applied Filters: ** Active not recruiting  Completed  Terminated  With Results  Interventional  Adult (18–64)  Older Adult (65+)**

# Table S2. Outcome event definitions

| Study | Death | Cardiovascular Death | Cardiac Death | Myocardial Infarction | Stroke | Major Bleeding | Stent Thrombosis |
| --- | --- | --- | --- | --- | --- | --- | --- |
| Bonaca, Marc P., et al. 2015 | Deaths will be subclassified by CV and non-cardiovascular primary cause. | CV death includes sudden cardiac death, death due to acute MI, death due to heart failure, death due to a cerebrovascular event, death due to other cardiovascular causes (eg, pulmonary embolism, aortic disease, cardiovascular intervention), and deaths for which there was no clearly documented non-cardiovascular cause (presumed CV death). | - | MI is diagnosed based on the Universal MI definition.^1^ | Stroke is defined as an acute episode of neurologic dysfunction attributed to a central nervous system vascular cause. Stroke should be documented by imaging (eg, CT scan or magnetic resonance imaging [MRI] scan). Evidence obtained from autopsy can also confirm the diagnosis. | TIMI^2^ Bleeding Classification. | - |
| Connolly, Stuart J., et al. 2018 | Deaths will be subclassified by CV and non-cardiovascular primary cause. | Defined as a death for which a definite non-cardiovascular cause (e.g. cancer) has not been identified. Uncertain causes of deaths are presumed to be CV unless proven otherwise. | - | The third universal definition of MI^3^. | Stroke is defined as the presence of acute focal neurological deficit thought to be of vascular  origin with signs and symptoms lasting ≥ 24 hours or to time of death. On the basis of clinical  symptoms or signs, and computerized tomography (CT) or MRI imaging. | ISTH^4^ major bleeding definition. | Definite stent thrombosis by angiographic and pathological confirmation. Probable stent thrombosis if occurred after intracoronary stenting . |
| Dadjou, Yahya, 2016 |  | ARC criteria^5^. | - | MI is diagnosed based on the Universal MI definition.^1^ | - | TIMI^2^ criteria and the  Bleeding Score^6^. | ARC criteria^5^. |
| Hahn, Joo-Yong, et al. 2018 | All deaths were considered cardiac unless a definite non-cardiac cause could be established. | - | - | Elevated cardiac enzymes (cardiac troponin or myocardial band fraction of creatine kinase) above the upper reference limit with ischaemic symptoms or electrocardiography findings indicative of ischaemia that was not related to the index procedure. | Stroke was defined as any non-convulsive focal or global neurological deficit of abrupt onset lasting more than 24 h or leading to death, which was caused by ischaemia or haemorrhage within the brain. | BARC^5^ type 3–5 bleeding | ARC criteria^5^. |
| Helft, Gérard, et al. 2016 | All deaths reported post-enrolment were recorded and adjudicated. Deaths were subclassified by CV and non-cardiovascular primary cause. CV death included sudden cardiac death, death due to acute MI, death due to heart failure, death due to a cerebrovascular event, death due to other CV causes (e.g. pulmonary embolism, aortic disease, CV intervention), and deaths for which there was no clearly documented non-cardiovascular cause (presumed CV death). | - | - | The third universal definition of myocardial infarction^3^. | Strokes were categorized as ischaemic or haemorrhagic depending on the results of cerebral imaging. | ISTH^4^/TIMI^2^ classification. | ARC criteria^5^. |
| Lee, Cheol Whan, et al. 2014 | All deaths were considered to have resulted from cardiac causes unless an unequivocal noncardiac cause could be established. | - | - | The diagnosis of acute MI was based on the universal definition of MI^1^. | Stroke, as detected by the occurrence of a new neurological deficit, was confirmed by a neurologist and imaging. | TIMI^2^ Bleeding Classification. | *ARC criteria^5^. |
| Mauri, Laura, et al. 2014 | All deaths^5^ are considered cardiac unless an unequivocal non-cardiac cause can be established. Specifically, any unexpected death even in subjects with coexisting potentially fatal non-cardiac disease (e.g. cancer, infection) should be classified as cardiac. | - | Cardiac death Any death due to immediate cardiac cause (e.g. MI, low-output failure, fatal arrhythmia). Unwitnessed death and death of unknown cause will be classified as cardiac death. This includes all procedure related deaths including those related to concomitant treatment.Death due to cerebrovascular disease, pulmonary embolism, ruptured aortic aneurysm, dissecting aneurysm, or other vascular cause. | Adapted from Global Task Force Universal Definition of MI^1^. | Cerebrovascular accident is defined as the occurrence of cerebral infarction (ischemic stroke) or intracerebral hemorrhage and subarachnoid hemorrhage (hemorrhagic stroke). | GUSTO^7^ criteria. | ARC^5^ criteria. |
| Park, Seung-Jung, et al. 2010 | All deaths were considered to be from cardiac causes unless an unequivocal noncardiac cause could be established. | - | - | Universal definition of MI^1^. | Stroke, as detected by the occurrence of a new neurologic deficit, was confirmed by a neurologist and on imaging. | TIMI^2^ Bleeding Classification. | *ARC criteria^5^. |
| Roe, Matthew T., et al. 2012 | - | CV death, death from documented cardiovascular cause or not clearly attributable to noncardiovascular causes. | - | Universal definition of MI^1^. | Rapid onset of new, persistent neurologic deficit lasting >24 h: “ischemic” or “hemorrhagic” (if imaging data available), or “uncertain cause” (if imaging data unavailable). | GUSTO^7^/TIMI^2^ classification. | - |
| Steg, P. Gabriel, et al. 2019 | All deaths reported post-randomization will be recorded and adjudicated. Deaths after patient withdrawal of consent will be recorded but not adjudicated. Deaths will be sub-classified by CV and non-CV primary cause. CV death includes sudden cardiac death, death due to acute MI, death due to heart failure or cardiogenic shock, death due to a cerebrovascular event, death due to other CV causes (e.g., pulmonary embolism, aortic disease, CV intervention), and deaths for which there was no clearly documented non- CV cause (presumed CV death). | CV death includes sudden cardiac death, death due to acute myocardial infarction, death due to heart failure, death due to a cerebrovascular event, and death due to other CV causes. |  | Third Universal MI definition^3^. | Stroke is defined as an acute episode of neurologic dysfunction attributed to a central nervous system vascular cause. Stroke should be documented by imaging (e.g., CT scan or magnetic resonance imaging [MRI] scan) showing an area of acute infarction compatible with the neurologic symptoms. Evidence obtained from autopsy can also confirm the diagnosis. | TIMI^2^ classification. | ARC criteria^5^. |
| Valgimigli, Marco, et al. 2012 | All deaths were considered to be of cardiovascular causes unless an unequivocal noncardiovascular cause could be established. |  |  | Universal definition of MI^1^. | Stroke, as detected by the occurrence of a new neurological deficit, was confirmed by a neurologist and on imaging, whereas the occurrence of a transient ischemic attack required hospitalization and clinical confirmation by a neurologist. | TIMI^2^ criteria and the BleedScore^6^. | ARC criteria^5^. |

*Definite stent thrombosis only.

MI=Myocardial Infarction; CV=Cardiovascular; GUSTO=Global Utilization of Streptokinase and TPA for Occluded arteries; BARC=Bleeding Academic Research Consortium; TIMI= Thrombolysis in Myocardial Infarction; ARC=Academic Research Consortium criteria; ISTH= the International Society on Thrombosis and Haemostasis.

# Table S3. Clinical Presentation at Enrollment

| Study Name | Stable CAD N/(%) | Unstable angina  (N) | NSTEMI  (N) | STEMI  (N) | Others  (N) | ACS Presentation at Enrollment  (N) | PCI indication N/ (%) |
| --- | --- | --- | --- | --- | --- | --- | --- |
| DAPT STUDY  (Mauri, Laura, et al. 2014) | 3752/(37.66%) | 1663 | 1543 | 1045 | 1958 | 4251 | 9961/(100%) |
| NCT00977938 |  |  |  |  |  |  |  |
| THEMIS  (Steg, P. Gabriel, et al. 2019) | 19220/ (100%) | - | | | | | - |
| NCT01991795 |  |  |  |  |  |  |  |
| COMPASS  (Connolly, Stuart J., et al. 2018) | 16574/(100%) | - | | | | | - |
| NCT01776424 |  |  |  |  |  |  |  |
| OPTIDUAL  (Helft, Gérard, et al. 2016) | 447/(32.27%) | 129 | 216 | 156 | 148 | 501 | 1385/(100%) |
| NCT00822536 |  |  |  |  |  |  |  |
| PEGASUS-TIMI  (Bonaca, Marc P., et al. 2015 ) | 21162/(100%) | - | | | | | - |
| NCT01225562 |  |  |  |  |  |  |  |
| DES-LATE  (Lee, Cheol Whan, et al. 2014) | 1967/(38.98%) | 1901 | 534 | 628 | 15 | 3063 | 5045/(100%) |
| NCT01186146 |  |  |  |  |  |  |  |
| PRODIGY  (Valgimigli, Marco, et al. 2012) | 507/(25.74%) | 365 | 450 | 648 | - | 1465 | 1970/(100%) |
| NCT00611286 |  |  |  |  |  |  |  |
| Dadjou, Yahya, 2016 | - | | | | | | 1010/(100%) |
| NCT02327741 |  |  |  |  |  |  |  |
| REAL-Late/ZEST Late  (Park, Seung-Jung, et al. 2010) | 1014/(37.54%) | 1102 | 289 | 296 | - | 1687 | 2701/(100%) |
| NCT00484926,NCT00590174 |  |  |  |  |  |  |  |
| SMART-DATE^$^  (Hahn, Joo-Yong, et al. 2018) | - | 675 | 690 | 826 | - | 2191 | 2191/(100%) |
| NCT01701453 |  |  |  |  |  |  |  |
| TRILOGY  (Roe, Matthew T., et al. 2012) | - | 2356 | 4887 | - |  | 7243 | Excluded |
| NCT00699998 |  |  |  |  |  |  |  |

Abbreviations: ACS: acute coronary syndrome; CABG: coronary artery bypass graft; CAD: coronary artery disease; PCI: percutaneous coronary intervention; STEMI: ST-elevation myocardial infarction; NSTEMI: non- ST-elevation myocardial infarction.

$ Data referred to patients assigned to clopidogrel.

# Table S4. Medical Therapy Used in Included Trials

| Study | Lipid-lowering agent (N) | ACE inhibitor or ARB (N) | Beta-blocker (N) | PPI (N) |
| --- | --- | --- | --- | --- |
| DAPT STUDY  (Mauri, Laura, et al. 2014) | - | - | - | - |
| NCT00977938 |  |  |  |  |
| THEMIS  (Steg, P. Gabriel, et al. 2019) | 17266 | 15114 | 14192 | 4901 |
| NCT01991795 |  |  |  |  |
| COMPASS  (Connolly, Stuart J., et al. 2018) | 15240⑀ | 11909 | 12278 | - |
| NCT01776424 |  |  |  |  |
| OPTIDUAL  (Helft, Gérard, et al. 2016) | 1300 | 1034 | 1105 | 669 |
| NCT00822536 |  |  |  |  |
| PEGASUS-TIMI  (Bonaca, Marc P., et al. 2015 ) | 19604 | 17030 | 17486 | - |
| NCT01225562 |  |  |  |  |
| DES-LATE  (Lee, Cheol Whan, et al. 2014) | 4150 | 2551 | 3308 | - |
| NCT01186146 |  |  |  |  |
| PRODIGY  (Valgimigli, Marco, et al. 2012) | 1803 | 1687 | 1638 | 738 |
| NCT00611286 |  |  |  |  |
| Dadjou, Yahya, 2016 | 993 | 522 | - | - |
| NCT02327741 |  |  |  |  |
| REAL-Late/ZEST Late  (Park, Seung-Jung, et al. 2010) | 2139 | 1236 | 1786 | - |
| NCT00484926,NCT00590174 |  |  |  |  |
| SMART-DATE^$^  (Hahn, Joo-Yong, et al. 2018) | 1980 | 1529 | 1583 | - |
| NCT01701453 |  |  |  |  |
| TRILOGY  (Roe, Matthew T., et al. 2012) | 6080⑀ | 5432 | 5635 | 1666 |
| NCT00699998 |  |  |  |  |

Abbreviation: ACE= Angiotensin-converting enzyme; ARB= Angiotensin receptor blocker; PPI= Proton-pump inhibitor.

⑀ all medications was statin except of two marked trials.

$ Data referred to patients assigned to clopidogrel.

# Table S5. Baseline patients’ characteristics

| Study | Prior Percutaneous Coronary Intervention (N) | Peripheral Artery Disease  (N) | Prior Myocardial infarction (N) | | Hypertension  (N) | | | Diabetes mellitus  (N) | Dyslipidemia  (N) | Current Smoker  (N) | Previous stroke/ TIA  (N) | Prior Coronary Artery Bypass Graft (N) | Multivessel Disease  N/(%) |
| --- | --- | --- | --- | --- | --- | --- | --- | --- | --- | --- | --- | --- | --- |
| DAPT STUDY  (Mauri, Laura, et al. 2014) | 3047 | 568 | 2118 | | 7445 | | | 3037 | - | 2432* | 324 | 1149 | - |
| NCT00977938 |  |  |  |  |  |  |  |  |  |  |  |  |  |
| THEMIS  (Steg, P. Gabriel, et al. 2019) | 11154 | 1687 | EXCLUDED | | 17776 | | | 19220 | 16753 | 2094 | EXCLUDED | 5537 | 11935/(62.10%) |
| NCT01991795 |  |  |  |  |  |  |  |  |  |  |  |  |  |
| COMPASS (Connolly, Stuart J., et al. 2018) | 9876 | 3297 | 11375 | | 12498 | | | 6083 | - | 3366 | 547 | 5290 | 10295/(62.12%) |
| NCT01776424 |  |  |  |  |  |  |  |  |  |  |  |  |  |
| OPTIDUAL  (Helft, Gérard, et al. 2016) | 366 | 79 | 241 | | 813 | | | 435 | - | 824* | 54 | 72 | 757/(54.66%) |
| NCT00822536 |  |  |  |  |  |  |  |  |  |  |  |  |  |
| PEGASUS-TIMI (Bonaca, Marc P., et al. 2015 ) | 17568 | 1143 | 21135 | | 16407 | | | 6806 | 16241 | 3536 | EXCLUDED | - | 12558/(59.34%) |
| NCT01225562 |  |  |  |  |  |  |  |  |  |  |  |  |  |
| DES-LATE  (Lee, Cheol Whan, et al. 2014) | 589 | - | 195 | | 2902 | | | 1418 | - | 1415 | 104 | - | 2463/(48.82%) |
| NCT01186146 |  |  |  |  |  |  |  |  |  |  |  |  |  |
| PRODIGY (Valgimigli, Marco, et al. 2012) | 358 | 246 | 528 | | 1414 | | | 477 | 1078 | 469 | 76 | 215 | 1292/(65.58%) |
| NCT00611286 |  |  |  |  |  |  |  |  |  |  |  |  |  |
| Dadjou, Yahya, 2016 | - | | | | 553 | | | 283 | 422 | 341 | 16 | - | - |
| NCT02327741 |  |  |  |  |  |  |  |  |  |  |  |  |  |
| REAL-Late/ZEST Late (Park, Seung-Jung, et al. 2010) | 336 | - | 96 | | 1540 | | | 704 | 1170 | 835 | 102 | - | 1300/(48.13%) |
| NCT00484926,NCT00590174 |  |  |  |  |  |  |  |  |  |  |  |  |  |
| SMART-DATE^$^ (Hahn, Joo-Yong, et al. 2018) | - | | | - | | 44 | 1080 | 602 | 541 | 856 | 91 | - | 988/(45.10%) |
| NCT01701453 |  |  |  |  |  |  |  |  |  |  |  |  |  |
| TRILOGY  (Roe, Matthew T., et al. 2012) | 2022 | 472 | 3168 | | 5820 | | | 2811 | 4270 | 1566* | EXCLUDED | 1115 | - |
| NCT00699998 |  |  |  |  |  |  |  |  |  |  |  |  |  |

* Current or recent cigarette smoker.

$ Data referred to patients assigned to clopidogrel.

# **Table S6 : P-Scores for the treatment regimens and outcomes in** subgroup analysis

| Treatment Regimen | P-Score ranking^a^ | | |
| --- | --- | --- | --- |
|  | MI | Stent thrombosis | Major bleeding |
| Rivaroxaban | 0.360 | 0.212 | 0.583 |
| Prasugrel | 0.989 | 0.933 | 0.402 |
| Ticagrelor60 | 0.405 | 0.309 | 0.224 |
| Ticagrelor90 | 0.449 | 0.488 | 0.097 |
| Clopidogrel | 0.730 | 0.856 | 0.695 |

P-Score ranking of efficacy and safety outcomes of various dual anti-thrombotic regimens for chronic coronary syndrome.

^a^The P-score represents the probability that each intervention is better than all the competing interventions, as derived from network point estimates and standard errors. MI, myocardial infarction.

# Figure S1. Diagram of network meta-analysis


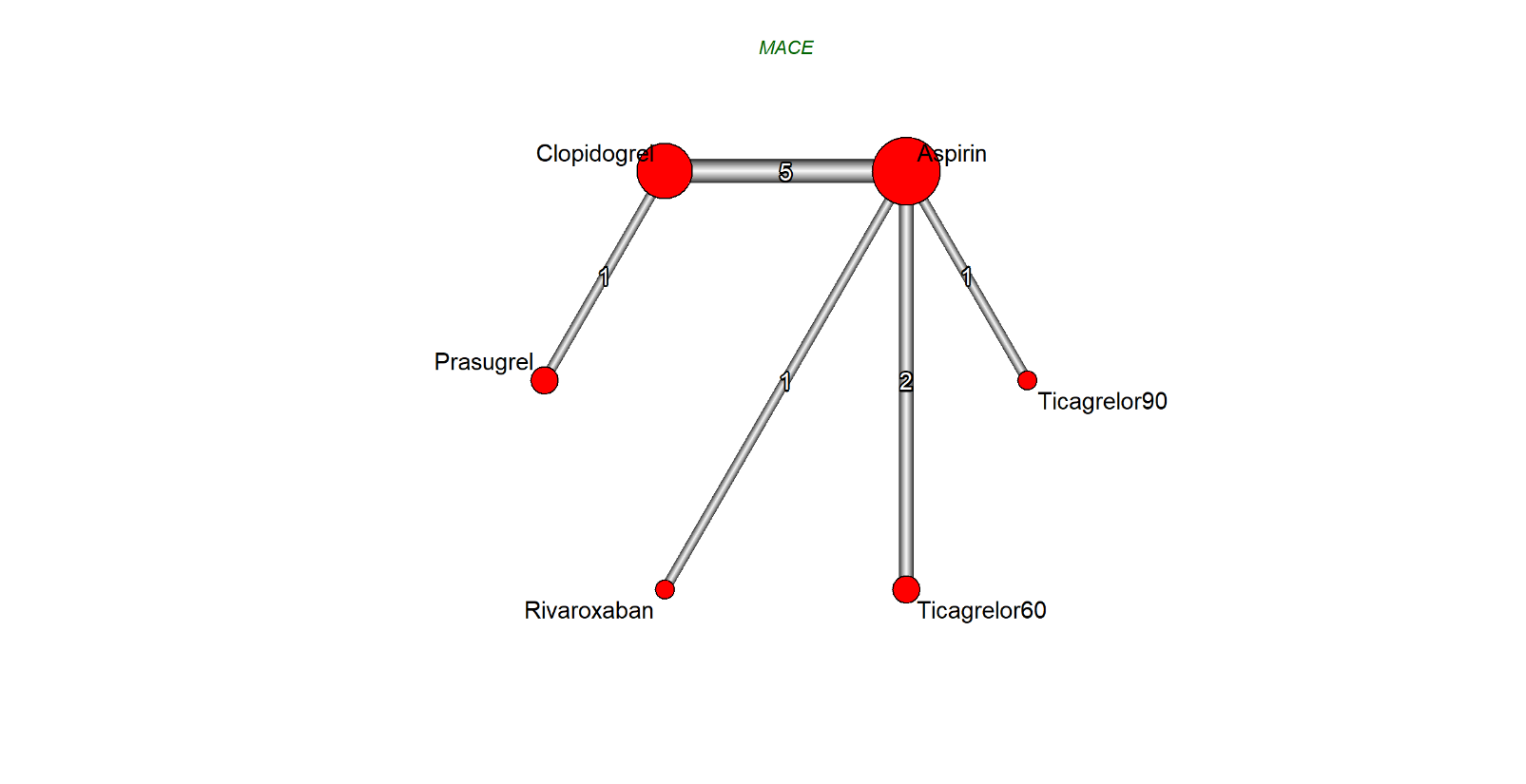

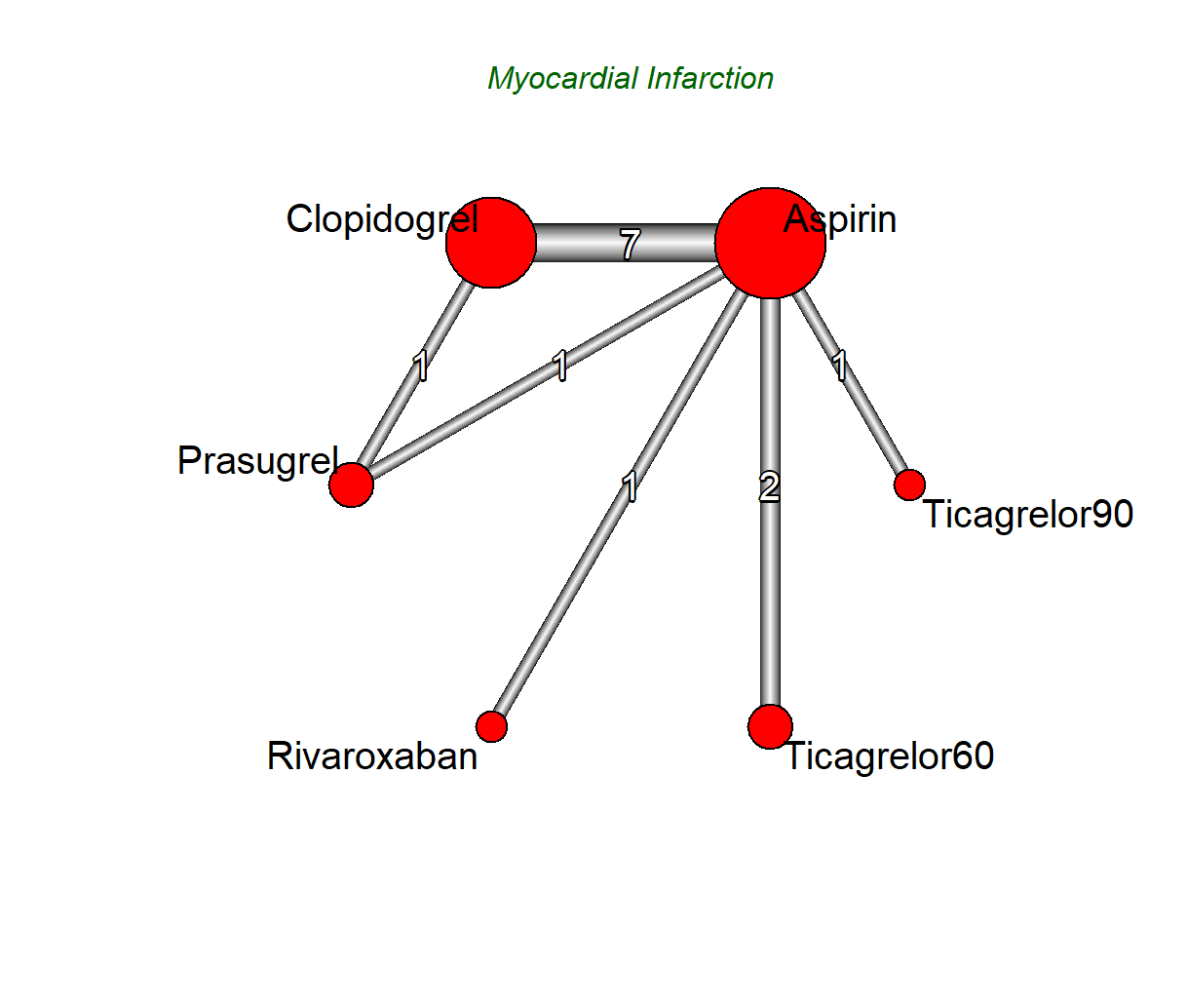


# Figure S2. Risk of bias assessment for randomized control studies, evaluated by the Cochrane Collaboration's Risk of Bias Tool


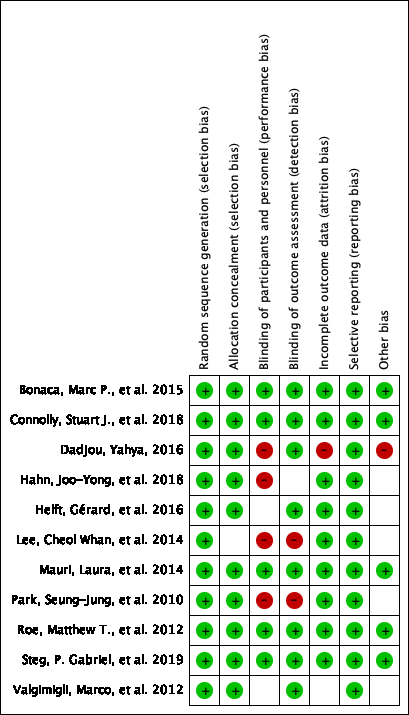


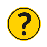

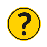

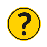

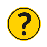

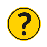

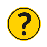

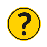

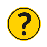

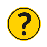

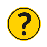


#

# Figure S3. Risk of bias graph : review authors' judgements about each risk of bias item presented as percentages across all included studies


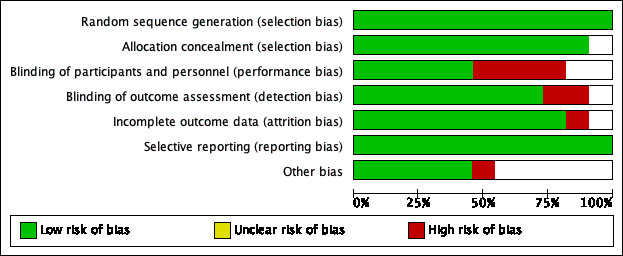


# Figure S4. myocardial infarction funnel plot and Egger's regression test


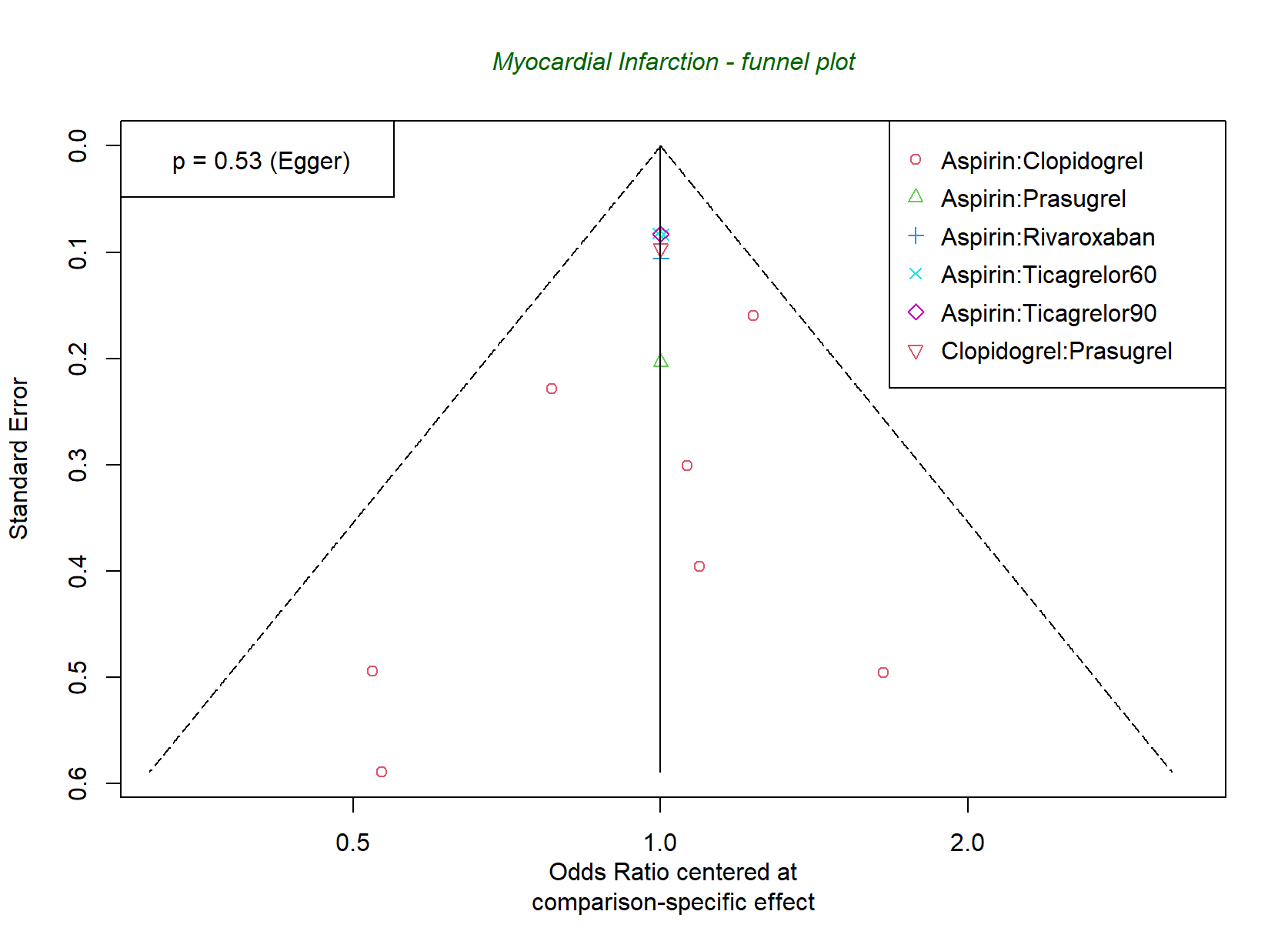


Egger’s test gave a P-value (2-tailed) = 0.53.

# References

1. Thygesen, Kristian, et al. "Universal definition of myocardial infarction." Journal of the American College of Cardiology 50.22 (2007): 2173-2195.‏
2. Rao, A. Koneti, et al. "Thrombolysis in Myocardial Infarction (TIMI) Trial—phase I: hemorrhagic manifestations and changes in plasma fibrinogen and the fibrinolytic system in patients treated with recombinant tissue plasminogen activator and streptokinase." Journal of the American College of Cardiology 11.1 (1988): 1-11.‏
3. ESC Committee for Practice Guidelines (CPG), et al. "Third universal definition of myocardial infarction." Journal of the American College of Cardiology 60.16 (2012): 1581-1598.‏
4. Schulman, S., C. Kearon, and Subcommittee on Control of Anticoagulation of the Scientific and Standardization Committee of the International Society on Thrombosis and Haemostasis. "Definition of major bleeding in clinical investigations of antihemostatic medicinal products in non‐surgical patients." Journal of thrombosis and haemostasis 3.4 (2005): 692-694.‏
5. Cutlip, Donald E., et al. "Clinical end points in coronary stent trials: a case for standardized definitions." Circulation 115.17 (2007): 2344-2351.‏
6. Serebruany, Victor L., and Dan Atar. "Assessment of bleeding events in clinical trials—proposal of a new classification." The American journal of cardiology 99.2 (2007): 288-290.‏
7. Gusto Investigators. "An international randomized trial comparing four thrombolytic strategies for acute myocardial infarction." New England Journal of Medicine 329.10 (1993): 673-682.‏
