# Supplementary figures and images for: Efficacy and safety outcomes of long-term anti-thrombotic treatment of chronic coronary artery disease: A systematic review and network meta-analysis
Source: Front Cardiovasc Med. 2023 Jan 9;9:1016390. doi: 10.3389/fcvm.2022.1016390 (PMC9868614; doi:10.3389/fcvm.2022.1016390)

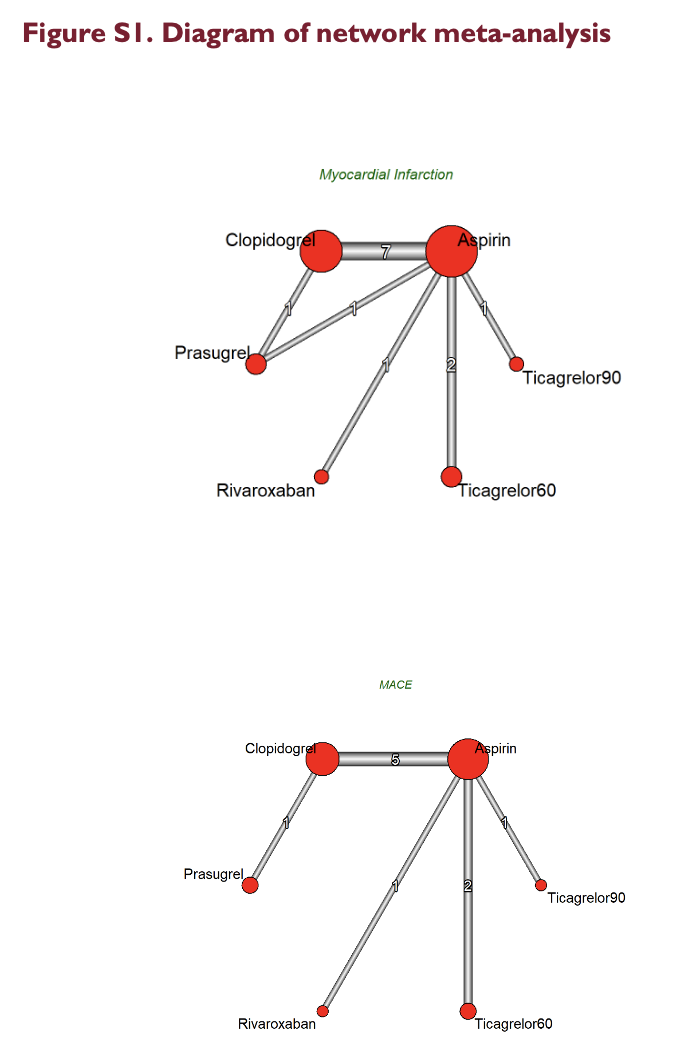

Supplement: Supplementary file 2 [file Image_1.PNG]

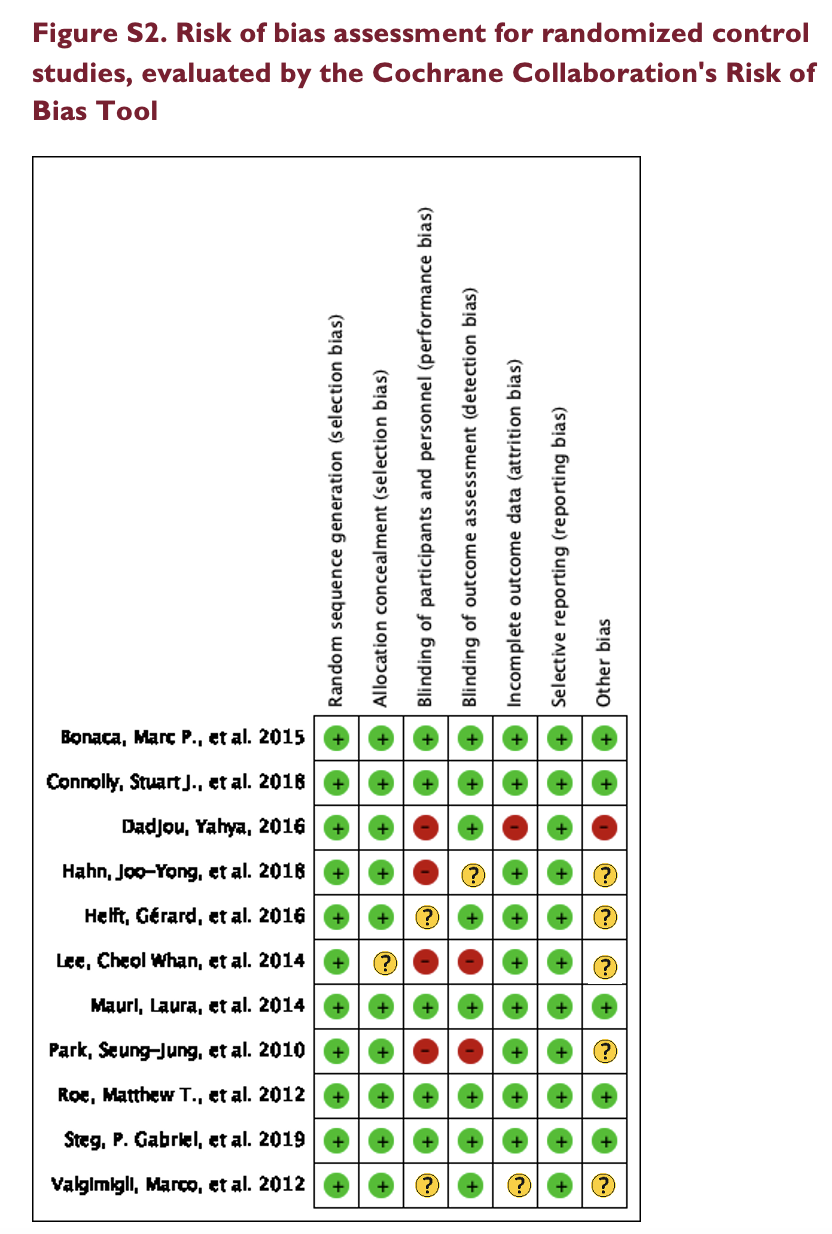

Supplement: Supplementary file 3 [file Image_2.PNG]

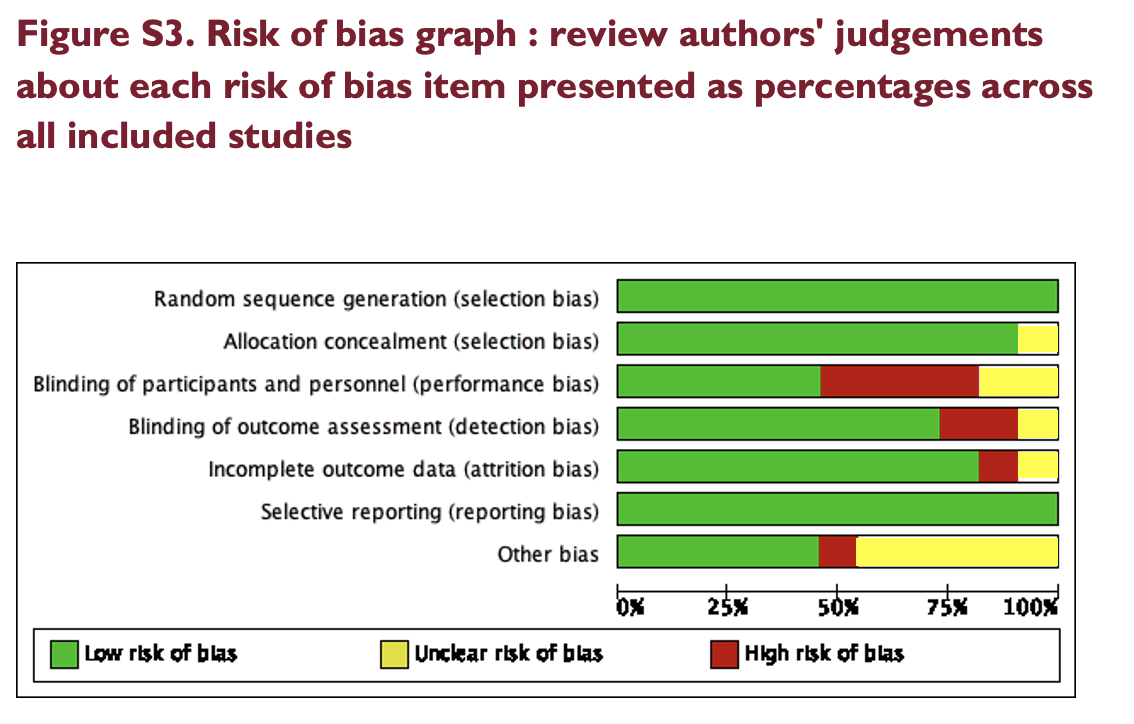

Supplement: Supplementary file 4 [file Image_3.PNG]

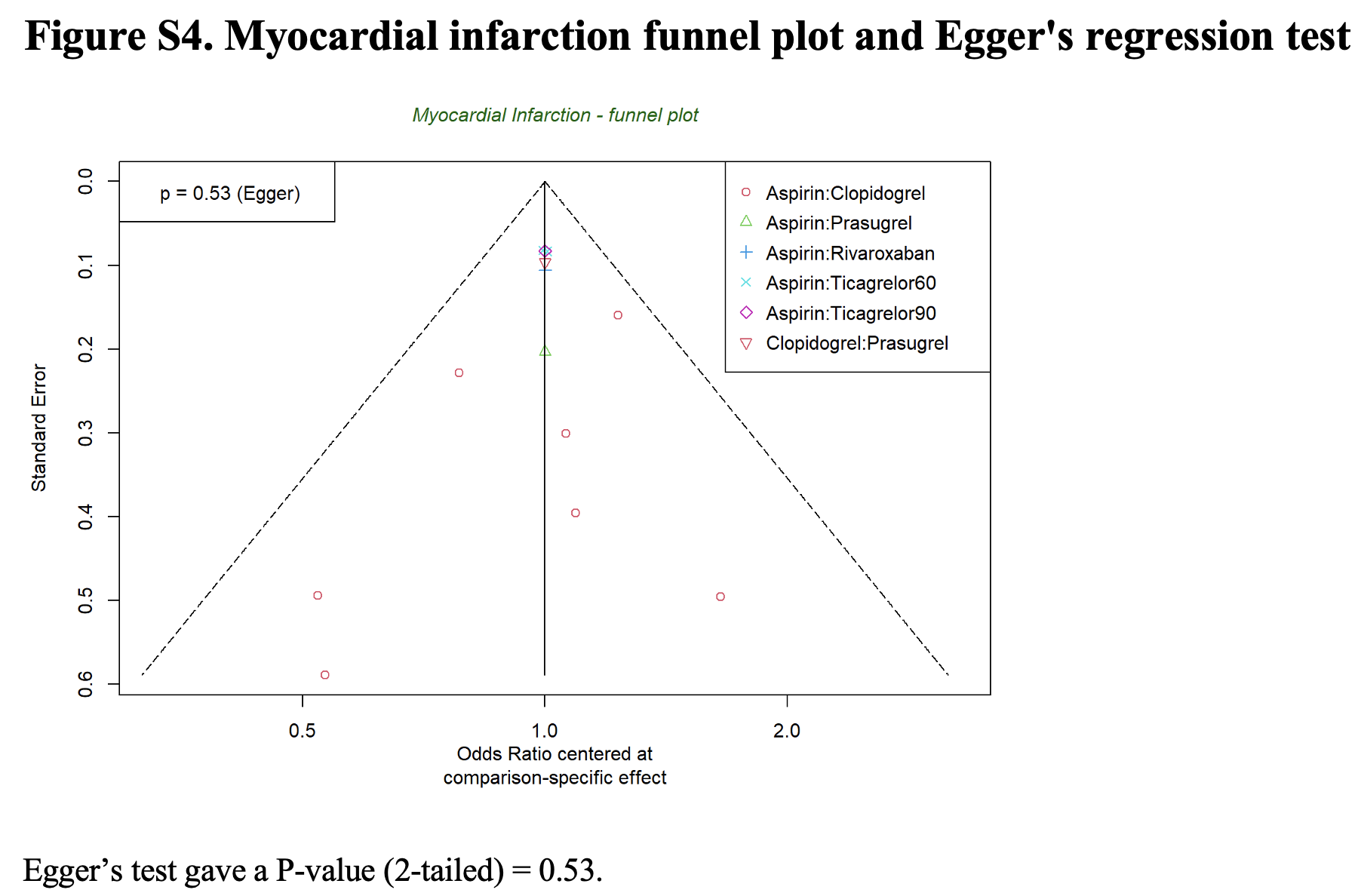

Supplement: Supplementary file 5 [file Image_4.PNG]
